# DRAW.IN.G.: A tool to explore children’s representation of the preschool environment

**DOI:** 10.3389/fpsyg.2022.1051406

**Published:** 2022-12-20

**Authors:** Sara Berti, Ada Cigala

**Affiliations:** Department of Humanities, Social Sciences and Cultural Industries, University of Parma, Parma, Italy

**Keywords:** drawings, interviews, preschool, environment, early childhood education and care centers, qualitative methods

## Abstract

The use of drawing as a research tool has often been the subject of debate in the field of developmental psychology, especially for the exploration of children’s meanings on a specific topic. Methodological limitations do emerge when using drawing in research, especially in preschool age. One of the main critical aspects concerns the lack of systematic and standardized coding methods that include clear and operationalizable categories to analyze the content of the drawings, and that associate a brief interview with the children aimed at avoiding misinterpretations. To bridge this gap, the present contribution introduces a new methodological tool named DRAW.IN.G. (DRAWing and Interview Grid), consisting of a specific procedure and a coding system that allow for a systematic investigation of implicit and explicit levels of children’s representation emerging *via* drawings and interviews. The specific topic investigated by DRAW.IN.G. is children’s representation of the preschool environment; the scarcity of studies on this issue, despite the importance of including children’s point of view in the design processes of educational spaces makes the tool particularly current and relevant to fill some gaps in research in the educational field. The DRAW.IN.G. coding system, developed on the basis of existing literature on the analysis of drawings, includes five main dimensions of children’s representation of the educational environment: physical, behavioral, relational, emotional and motivational dimensions, articulated in 18 macro-categories and 90 categories that make up the scoring grid. To assess the validity of the method, a first application was conducted with a sample of 262 children (141 males, 121 females; mean age = 55.78 months; SD = 11.10; range 37–77 months) from five Italian preschools. Categorical inter-rater reliability of two independent raters showed good to excellent agreement for the categories of the grid, indicating their appropriateness and clarity. The validation study indicated the potential of the method, also revealing some critical aspects to be considered. Both methodological and practical implications are discussed.

## Introduction

Drawing seems to be a feasible and enjoyable activity for most children, also representing a relatively easy way to obtain information about children’s experience (King, 1995). However, the use of drawing as a research tool is controversial and has often been the subject of debate in the field of developmental psychology; although the gains of the method are recognized, many methodological limitations do emerge when conducting research through drawing.

Among the advantages, the literature indicates children’s familiarity with the drawing activity, the provision of access to the thoughts and points of view of even the youngest children, the opportunity to investigate contents that are not fully accessible to awareness, and the slow processing of such contents due to the time needed to actually make the drawing (Dockett and Perry, 2005; Einarsdóttir, 2007). Furthermore, it emerged that many children are more inclined to complete drawing tasks than answer questions on a topic (Lewis and Greene, 1983) and provide a more detailed and emotional narrative if they are asked to draw first (Driessnack, 2005; Katz and Hamama, 2013).

Among the disadvantages, the literature indicates the fact that such a form of expression may be idiosyncratic, personal and liable to misinterpretation, and that children might not provide an original work as they could imitate the drawings of others (Thomas and Jolley, 1998; Bland, 2012). Such critical aspects are emphasized in the preschool age; while around the age of 7–9 year-old children develop a graphic language that includes specific symbols and rules of spatial organization, increasing their ability by 9–11 years (Barraza, 1999; Walker and Walker, 2007), in preschool age graphic skills are not yet developed and the drawings provided by children are often partially or totally incomprehensible to adults.

Because of these issues, the need to combine drawing with other tools that can integrate information collection has emerged (Thomas and Jolley, 1998; Bland, 2012); in particular, verbal interaction and discussion with the authors of the drawings on their production seem to be useful aids with which to avoid interpretative errors, especially on the analysis of the content (Yuen, 2004; Darbyshire et al., 2005). The integration of drawings and interviews would thus be effective, also considering that drawings support the expression of implicit meanings, complementing the explicit information obtained from interviews (Crook, 1985; Thomas and Silk, 1990; Farokhi and Hashemi, 2011). The development of standardized methods integrating these two methods thus emerges as a need for research in the field of educational psychology.

As for the aims that can be pursued in research using children’s drawings, some authors have stated that drawings are usually analyzed for one (or more) of the following purposes: personality assessment; evaluation of current emotional states; evaluation of personal significance of topic depicted; assessment of intelligence or developmental level; and assessment of possible neurological impairment (Thomas and Jolley, 1998). In relation to this, another critical issue highlighted by the literature is that, while drawings have often been used in the field of clinical psychology to assess personality traits (the first of the aforementioned purposes) or the cognitive development of children (fourth and fifth purposes), little has been investigated about drawings in relation to the second and the third aims, concerning children’s emotions and meanings in relation to a specific theme (second and third purposes), particularly in the field of education ( Einarsdóttir, 2007; Sharp, 2009; Bland, 2012). Nevertheless, drawings seem to be a proper tool with which to explore children’s significance on a specific topic; through drawings, in fact, children provide insights into their feelings and thoughts about that topic by reflecting an image of their own mind (Crook, 1985; Thomas and Silk, 1990).

The knowledge of children’s meanings on topics related to their development contexts should be incentivized, as it allows us to include their point of view in educational processes, choices and proposals. The relevance of involving children’s voices and perspectives in research has received an increasing attention in recent years (Hill, 2006; Roberts, 2017), as children are considered “beings” rather than “becomings” (Davies, 2014; Gutierrez-Vicario, 2021); the exploration of their perspective is thus fundamental to understand their life words, as they are seen as social constructors and active participants in the processes of their own experiences and learning (Mayall, 2000; Smith, 2007; Harcourt and Einarsdottir, 2011).

The importance of listening to children is supported by several international statements. Article 12 of the Convention on the Rights of the Child (The United Nations, 1989) indicates that children have the right to express their views on matters that affect their lives and that such views should be taken into account by adults. The General Comment No. 7 highlights the importance for young children to participate in decision-making processes concerning their development by expressing their own perspective (The United Nations, 2005). Such an attitude is also encouraged by the Child-Centered Approach promoted by UNICEF (2018), which encourages adults to listen to children’s voices about their concerns and thoughts, and to let them participate actively in the educational processes.

The above considerations allow to emerge two main gaps in the literature on the use of drawing as a research tool in educational psychology: on the one hand, the need to develop standardized methods that integrate drawings and interviews, on the other, the need to explore its use for the investigation of children’s meanings on specific topics. To bridge these gaps, the present contribution aimed at the initial development and standardization of a new systematic method of analysis of drawing associated with an interview, named DRAW.IN.G. (DRAWing and INterview Grid).

In particular, the DRAW.IN.G. tool aims to identify some elements that can help to approach children’s meanings about their experience of the Early Childhood Education and Care (ECEC) environment. The importance of taking into account children’s visions about their ECEC spaces has been deepened in a recent review on the topic (Berti et al., 2019) which underlines that there are still too few studies aimed at understanding how children perceive the physical environment of their ECEC services. Nevertheless, children’s meanings on such a topic are particularly relevant because they are the first “users” of ECEC spaces and the actors for whom such spaces are designed and realized. Taking into account their point of view would allow us to create environments that respond to their real needs and to make them active participants in the processes involved with their own development (Nah and Lee, 2016; Berti et al., 2019).

From the aforementioned literature review it also emerged that drawings and interviews were the main tools used to obtain children’s perceptions on the ECEC environment, but only two of the identified studies combine the use of both tools (Nah and Lee, 2016; Botsoglou et al., 2017). Nonetheless, the integration of drawing with the interview is a particularly suitable method to investigate the point of view of children related to the issues that affect their daily experiences, involving them directly, and that are significant for them also on an affective level. Drawings and interviews thus seem to be particularly useful to explore such issues, as through them children seem to provide indications about their relationship with the world and with other things, also expressing their emotions and thoughts (Farokhi and Hashemi, 2011). Both implicit and explicit aspects of their representation of ECEC spaces would then be explored through the DRAW.IN.G. tool. It should be clarified that for the purposes of this study, the definitions of implicit and explicit refer essentially to the way in which the data are collected: the implicit aspects are in fact deduced from the characteristics of the drawing, intended as a spontaneous action that also includes projective phenomena and knowledges not always aware and accessible to the authors of the drawing, while the explicit aspects are deduced from the interview, and refer to the motivations that are expressed verbally, implying an explicit awareness of the children.

The aim of the present article is to present the DRAW.IN.G. tool as new systematic method of analysis of drawing associated with an interview to explore children’s experience of their ECEC environment. Due to the methodological nature of the present study, the Method section consists of the presentation of the tool, its aim and target, the procedure for its administration, the description of the coding phase and of all the categories included in the coding system, and the possible analyses that can be carried out on the data collected through it. The Result section instead consists of the description of the first preliminary administration of the tool, including the characteristics of the participants, the description of analyses, also to assess the reliability of the tool, and the main results emerging from the collected data.

An in-depth description of the above-mentioned preliminary administration can be found in a recent article by Berti et al. (2022). It should be specified that, while the published study aimed at conducting a detailed investigation of children’s representation of ECEC spaces, by describing in depth the data collection and the emerging results, the present paper aims to describe the tool used to get such data, by presenting the procedure, the coding system, the included categories and the feasibility of the instrument. The published study aimed at providing data on children’s experience, while the present one aimed at providing a new methodological tool to the scientific community. Thus, in the present article the results emerging from the preliminary study are described only briefly, referring to Berti et al., 2022 for further information.

## Materials and methods

### Aim and target

The DRAW.IN.G. tool is aimed at a systematic investigation of children’s perception of the preschool environment by exploring implicit and explicit levels of representation emerging through drawings and interviews. In particular, the experience of space is conceptualized through five different dimensions: physical, behavioral, relational, emotional and motivational. Four dimensions (physical, behavioral, relational, emotional) connoted the implicit level and are investigated by means of drawings; one dimension (motivational) connoted the explicit level and is investigated by means of interviews. The method is addressed to children aged 3 to 6 years old who attend ECEC centers.

The *physical dimension* refers to the physical characteristics of the space, including which place is chosen, if it is indoors or outdoors, if it specific or generic, if architectural elements or furnishings are represented. The *behavioral dimension* refers to behaviors acted out in the space, including playing alone, playing with others, learning, observing nature, privacy moments, transitions from one space to another, eating, sleeping or going to toilet. The *relational dimension* refers to relationships that occur in space, including if people is represented, who is represented, which configuration of people is represented, which position people occupy in the drawing. The *emotional dimension* refers to emotions that connote the space, including by the representation of emotional states and archetypical elements, the use of colors and the position of the drawing in the sheet. The motivational dimension refers to the motivations stated on the choice of the represented space, including playing, learning, observing nature, having relationships, having moments of privacy, having a connection between indoor and outdoor spaces, having continuity between ECEC center and family, aesthetical reasons and functional reasons.

### Procedure

Having obtained informed consent for each participant, data collection takes place in a single day for each participating class. Each of the two tools-drawing and interview-is proposed at two different times and concerned two different procedures. The drawing activity, carried out simultaneously by all the children in the class, takes about half an hour; each interview, carried out with each child individually, takes about 5 min. The three phases of the procedure are described below, and their main characteristics are summarized in the Appendix.

#### Preliminary phase

As a preliminary phase, the researcher should introduce him/herself and the research to the class, specifying his/her role, the purpose of the research, the demand about the task, also trying to obtain informal consent from the children to participate in the study. For example, the researcher should say: “*Hi kids, I’m John and I’m a researcher, in other words, an inquisitive person trying to understand a few things*” (presentation of the researcher) “*Today I would like to understand which are the favorite places of children in their schools, and when I understand it I can write a book so others can find out about my discovery!*” (presentation of the research) “*Your school is made up of many places, some indoor others outdoor, so I will ask you to make a drawing on the place that you like the most here at school. You can draw any place you like, and you can also draw yourself or your teachers or other children or other people, as you like. When you have finished the drawing, I will ask you what you have drawn, so I can understand better*” (presentation of the task) “*Would you make a drawing for my book, so I can figure out what your favorite place is here at school?*” (asking for informal consent).

#### Drawing phase

After the preliminary stage, the researcher should introduce the drawing activity. Drawings should be made individually, to avoid the risk of imitation and copy among children. Each child should be provided with a blank sheet of A4 paper and each small group or child should be equipped with markers of various colors. It is important that all the main colors are present, both cold and warm: red, yellow, blue, orange, green, violet, pink, brown, gray, black.

When children are ready, the researcher should reiterate the task, by using this formula: *“Please draw the place where you like to stay the most when you are here at school.”* This formula has been identified starting from some preliminary studies conducted with preschoolers (Berti, 2021). Starting from these studies, it was decided to focus on the task concerning the preferred place, and not the school in general for different reasons. First, because the aim was to grasp children’s personal experience, thoughts, meanings and emotions about their ECEC spaces, and not an objective representation of the environment. Secondly, because preliminary studies have shown that the more general task “*Please draw your school*” seemed too generic, excessively complex and difficult to grasp for children of this age.

The activity should take all the time required; this is usually about half an hour. When a child has finished his/her drawing he/she can go to the researcher, who can start the interview phase. The presence of two researchers would be preferable, so that one researcher (or in his/her absence, the teacher) stays in the classroom with the children who are still finishing the drawing while the other one moves with one child at a time in the area dedicated to the interview.

#### Interview phase

**Figure 1:**
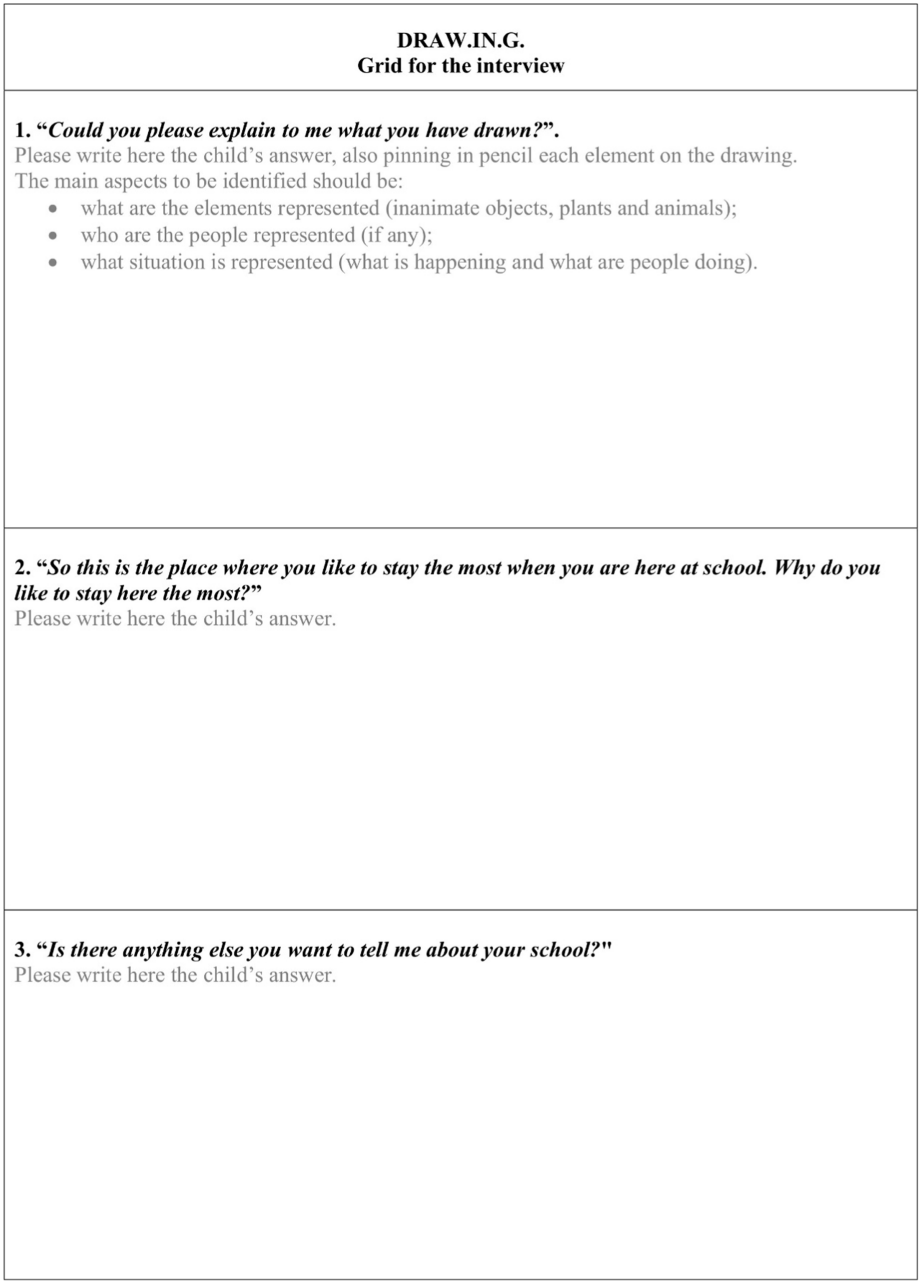
Interview grid.

Immediately after the drawing activity, the researcher should interview each child individually on the basis of an interview grid (see Figure 1). It would be important for the interview to take place in a room separate from the class, to promote the concentration of the children and the intimacy of the moment. The interview consists of three main steps.

Firstly, the researcher should understand what the child has drawn by asking: “*Could you please explain to me what you have drawn?*.” During this first step of the interview, the researcher should identify what are the elements represented (inanimate objects, plants and animals), who are the people represented (if any) and what situation is represented (what is happening and what are people doing). If the child does not explain some of these aspects, the researcher should ask for them indicating the elements on the drawing: *“What is this?”/“Who is this?”/“What is happening?”/“What are people doing?.”* The researcher should also ask other questions to better understand the child’s graphic representation, being careful not to condition the child by suggesting the answers, for example asking *“Who is this?”* rather than *“Who is this woman”* (he/she may not be a woman) or *“Is this your teacher?”* (it may not be). Given the young age of children, such an involuntary suggestion could generate a desirability bias in children’s responses because of their desire for approval.

Secondly, the researcher should ask each child the motivation for his/her preference, by asking *“So this is the place where you like to stay the most when you are here at school. Why do you like to stay here the most?.”* Also in this second step, the researcher should also ask other questions to better understand the motivation, being careful not to condition the child by suggesting reasons like for example: *“Do you like to stay here because there are other children?”*

Finally, the researcher should ask the child one last question to allow him/her to express other things about his/her experience at school, beyond what is specifically required by the task, using this formula: *“Is there anything else you want to tell me about your school?.”* This last question also has a debriefing function, allowing the child to relax and tell what he/she wants or say, generating a short chat before finishing the interview.

To conclude the interview, the researcher should thank the child for his/her drawing, telling him/her that it was very useful for his/her research and will be included in his/her book.

The activity should take all the time needed; this is usually about 5 min. All the interviews should be video-and audio-recorded to fill the interview grid in a later time. The video should frame the drawing while the child is explaining what he/she has drawn, so that the various elements defined during the first part of the interview can be identified and pinned later as well. The audio should record all the interview, so that a verbatim transcription of the child’s answer should be made later. If there is no possibility to record audio video, the identified elements can be pinned in pencil on the drawing itself and the child’s answers should be indicated in the interview grid by the researcher during the interview.

At the end of the data collection, the materials collected for each child will be the drawing with indication of each represented element, the interview grid and (if any) the audio video recording.

#### Preliminary screening

Preliminary to the application of the coding system, the children’s drawings should be screened in order to select only those relevant to the purposes of the research. Due to their early age, some children may not have understood the task and may have made a drawing that is not relevant to their favorite school space. A drawing is intended as “not relevant” when its content does not correspond to the task of drawing, a condition that can be ascertained during the interview: if the child explains that he has drawn something different from the space he likes most at school, his/her drawing is considered not relevant and must be excluded from the analysis. The evaluation of not relevant. Drawings, given its simplicity, can be carried out by the researcher himself during the interview. Examples of not relevant drawings are reported as Supplementary material.

### Coding

#### Draw.IN.G. coding system

The DRAW.IN.G. coding system was developed specifically for the systematic analysis of implicit and explicit levels of children’s representation of ECEC environments emerging through drawings and interviews, as drawings provide information about physical, behavioral, relational and emotional dimensions of space representation (implicit level) and interviews provide information about motivations for preferences (explicit level).

The definition of the macro-categories and categories was based on the researchers’ experience concerning the investigation of ECEC spaces (Berti et al., 2019; Berti, 2021) and the existing literature on the analysis of drawings, as described in the following sections. Some categories were identified on the basis of what the children had actually drawn (bottom-up process) while others were defined on the basis of literature (top-down process). The specific processes for the identification of the categories within each macro-category and the references related to the top-down processes are reported in Tables 1–5.

**Table 1:** Physical dimension: macro-categories and categories included in the DRAW.IN.G. grid.

Dimension	Macro-categories	Researcher’s question	Categories	Process^1^ (References)^2^
Physical	PHY_Space	Which school space is represented?	Outdoors	Bottom-up
			Class	
			Common spaces	
			All the school	
			Eating space	
			Sleeping space	
			Toilet space	
			Other	
	PHY_Specificity	It is a specific space or a generic space?	Specific	Top-down (Berti et al., 2019)
			Generic	
			Specific in a general context	
	PHY_Location	It is an indoor space or an outdoor space?	Indoor	Top-down (Berti et al., 2019)
			Outdoor	
			Both	
	PHY_Architecture^3^	Which architectural elements are represented?	None	Top-down (Markham, 1954)
			Walls	
			Floors	
			Ceilings/roof	
			Doors	
			Windows	
			Chimney	
			Fence	
	PHY_Furnishings^3^	Which furnishings are represented?	Indoor furnishings	Top-down (Berti et al., 2019)
			Outdoor furnishings	

1 Process of identification of the categories: Bottom-Up or Top-Down.

2 References related to Top-Down processes of identification of the categories.

3 Macro-category including non-mutually exclusive categories.

**Table 2:** Behavioral dimension: macro-categories and categories included in the DRAW.IN.G. grid.

Dimension	Macro-categories	Researcher’s question	Categories	Process^1^
Behavioral	BEH_Behavior	What behaviors were represented?	Playing alone	Bottom-up
			Playing with others	
			Learning moment	
			Observation of nature	
			Privacy moment	
			Not specified	
			Transition or wait	
			Eating moment	
			Sleeping moment	
			Toilet moment	

1 Process of identification of the categories: Bottom-Up or Top-Down.

**Table 3:** Relational dimension: macro-categories and categories included in the DRAW.IN.G. grid.

Dimension	Macro-categories	Researcher’s question	Categories	Process^1^ (References)^2^
Relational	REL_Representation	Are people represented?	People represented	Top-down (Bombi et al., 2007)
			People not represented	
	REL_Who^3^	Which people are represented	Child him/herself	Bottom-up
			Friends	
			Teachers	
			Familiars	
	REL_Configuration	Which configuration of people is represented?	No one	Bottom-up
			Only self	
			Only friends	
			Only teachers	
			Only family members	
			Self&Friends	
			Self&Teachers	
			Self&Family members	
			Self&Friends&Teachers	
	REL_Position_horizontal	In which horizontal portion of the paper are people represented?	Left	Top-down (Federici, 2007)
			Center	
			Right	
	REL_Position_vertical	In which vertical portion of the paper are people represented?	Top	Top-down (Federici, 2007)
			Center	
			Bottom	

1 Process of identification of the categories: Bottom-Up or Top-Down.

2 References related to Top-Down processes of identification of the categories.

3 Macro-category including non-mutually exclusive categories.

**Table 4:** Emotional dimension: macro-categories and categories included in the DRAW.IN.G. grid.

Dimension	Macro-categories	Researcher’s question	Categories	Process^1^ (References)^2^
Emotional	EMO_Climate	What emotional climate is represented?	Positive	Top-down (Bombi et al., 2007)
			Negative	
			Mixed	
			Neutral	
			Not represented	
	EMO_Archetypes^3^	What archetypical elements are represented?	Land line	Top-down (Crotti and Magni, 2011; Serraglio, 2011)
			Skyline	
			Sun	
			Moon	
			Trees	
			Flowers	
			Rainbow	
			Animals	
			Monsters	
	EMO_Colors_tone	What color tone is most represented?	Warm colors	Top-down (Lüscher et al., 1976)
			Cold colors	
			Both	
	EMO_Colors_variety	How many colors were used?	One color	Top-down (Crotti and Magni, 2011; Serraglio, 2011)
			Up to four colors	
			More than four colors	
	EMO_Position_horizontal	What horizontal portion of the paper does the drawing occupy?	Left	Top-down (Federici, 2007)
			Center	
			Right	
	EMO_Position_vertical	What horizontal portion of the paper does the drawing occupy?	Top	Top-down (Federici, 2007)
			Center	
			Bottom	

1 Process of identification of the categories: Bottom-Up or Top-Down.

2 References related to Top-Down processes of identification of the categories.

3 Macro-category including non-mutually exclusive categories.

**Table 5:** Motivational dimension: macro-categories and categories included in the DRAW.IN.G. grid.

Dimension	Macro-categories	Researcher’s question	Categories	Process^1^
Motivations^2^	MOT_Reason	What kind of reason does the child express for his preferences regarding space?	Playing	Bottom-up
			Learning	
			Observation of nature	
			Relationships	
			Privacy	
			Indoor/outdoor connection	
			Continuity with family	
			Aesthetical reasons	
			Functional reasons	
			Others	

1 Process of identification of the categories: Bottom-Up or Top-Down.

2 Macro-category including non-mutually exclusive categories.

Three independent researchers (two students engaged in master’s degree theses in psychology and a Ph.D. student in psychology) analyzed 120 drawings in order to identify the categories to be defined through the bottom-up process. Each researcher analyzed 40 drawings identifying categories that should answer to the questions related to each macro-category, then all the researchers compared and discussed their categories, finding an agreement on the final categories to be included in the scoring grid. The questions related to each macro-category and the identified categories are reported in Tables 1–5.

The final version of coding system includes 18 macro-categories and 90 categories for the physical, behavioral, relational and emotional dimensions (implicit level) and motivations (explicit level). Some included categories are mutually exclusive while others are not, as specified in the following section. The coding system for each dimension is reported in Tables 1–5. Examples of drawings for each category are reported as Supplementary material.

##### Physical dimension: Macro-categories and categories

The physical dimension refers to the physical characteristics of the space and include five main macro-categories: *PHY_Space,* referring to the school space represented by the child, *PHY_Specificity*, referring to the fact that the space represented was specific or generic; *PHY_Location*, referring to the fact that the space represented was indoors or outdoors; *PHY_Furnishings*, referring to the representation of indoor or outdoor furnishings; *PHY_Architecture*, referring to the representation of the school building.

*PHY_Space* includes eight mutually exclusive categories: *outdoors* indicates the representation of only outdoor spaces, such as gardens or playgrounds; *class* indicates the representation of the children’s only class; *common spaces* indicates the representation of spaces shared by children of different classes, such as the entrance, corridors and halls; *all the school* indicates the representation of the school as a whole, such as the representation of the school building and the surrounding garden; *eating space* indicates the space where children eat; *sleeping space* indicates the space where children sleep; *toilet space* indicates the space where children go to the toilet; *other* signifies other school spaces not mentioned in the previous categories.

*PHY_Specificity* includes three mutually exclusive categories: *specific* indicates the representation of a specific space, such as the doll’s corner for the indoors or the swings for the outdoors; *generic* indicates the representation of a generic space, such as the whole class or the entire garden; *specific in a general context* indicates the representation of a specific favorite space within a larger general context, such as the representation of the doll’s corner within the representation of the whole class, or the representation of the swings within the representation of the entire garden.

*PHY_Location* includes three mutually exclusive categories: *outdoors* indicates the representation of only outdoor spaces; *indoors* indicates the representation of only outdoor spaces; *both* indicates the representation of both indoor and outdoor spaces. *PHY_Furnishings* includes three non-mutually exclusive categories: *indoor furnishings*, *outdoor furnishings* and *both*.

*PHY_Architecture* includes eight non-mutually exclusive categories: *none* indicates that the representation does not include architectural elements of the school building, while the other seven categories indicate the representation of the elements defined by their name: *walls, floors, ceilings/roof, doors, windows, chimney, fence*. Such categories were extrapolated from the House Drawing test (Markham, 1954).

##### Behavioral dimension: Macro-categories and categories

The behavioral dimension refers to behaviors acted out in the space and include one variable named *BEH_Behavior* which is defined by 10 mutually exclusive categories: *Playing alone* refers to the representation of the child playing alone in the space, *Playing with others* refers to the representation of the child playing with other people in the space, *Learning moments* refers to the representation of the child reading writing, drawing or being involved in other activities related to the acquisition of academic skills*, Observation of nature* refers to the representation of the child observing plants, animals or other natural elements*, Privacy moments* refers to the representation of the child during a private moment, such as resting on a bench or taking refuge in a shelter*, Transition or wait* refers to the representation of moments when the child is transiting from one space to another, such as from home to school, or is waiting, as waiting for his mum at the end of the school day*, Eating moment, Sleeping moment, Toilet moment* refer to the representation of the child during eating, sleeping or toilet routines*; Not specified* refers to the representation of a situation that the child does not define (for example, when the child answers “I do not know” or “Nothing” to the question “What are you doing here in the garden?”).

##### Relational dimension: Macro-categories and categories

The relational dimensions refers to relationships that occur in space and include five macro-categories: *REL_Representation*, referring to whether the relationships are represented or not, *REL_Who*, referring to which people is represented; *REL_Configuration*, referring to the configuration of people, *REL_Position_horizontal, and REL_Position_vertical,* referring to the horizontal and vertical position of the representation of people on the sheet.

*REL_Representation* includes two mutually exclusive categories: People represented or people not represented. *REL_Who* includes four non-mutually exclusive categories, referring to the representation of the *child him/herself, friends, teachers, familiars. REL_Configuration* includes nine mutually exclusive categories, referring to the representation of *No one, Only Self, Only Friends, Only Teachers, Only Family members, Self&Friends, Self&Teachers, Self&Family members, Self&Friends&Teachers.*

*REL_Position_horizontal* includes three mutually exclusive categories defined by dividing the surface of the sheet into three equal parts and verifying in which of these parts the largest number of people or most of the body of people is placed: *left, center or right*. Similarly, *REL_Position_vertical* include the three mutually exclusive categories: *top, center and bottom*. Such categories were based on Walker and Walker (2007).

##### Emotional dimension: Macro-categories and categories

The emotional dimension refers to the emotions that connote the space and include six macro-categories: *EMO_Climate*, referring to the representation of emotions through facial expressions; *EMO_Archetypes*, referring to the representation of archetypical elements; *EMO_Colors_tone* and *EMO_Colors_variety*, referring, respectively, to the use of a prevalent colors tone and the use of few or many colors; *EMO_Position_horizontal* and *EMO_Position_vertical*. referring to the horizontal and vertical position of the graphical representation on the sheet surface.

*EMO_Climate* includes five mutually exclusive categories defined by the facial expression of the child when he/she has depicted himself/herself in the drawing: *positive, negative, mixed, neutral, not represented*. Such categories were based on Bombi et al. (2007). *EMO_Archetypes* includes 10 non-mutually exclusive categories based on the presence in the drawing of the archetypical elements identified by Crotti and Magni (2011) and Serraglio (2011): *Land line, Sky line, Sun, Moon, Trees, Flowers, Rainbow, Animals, Monsters*. *EMO_Colors_tone* includes three mutually exclusive categories defined by the use of a prevalent tone in the drawing: *warm, cold or both*. *EMO_Colors_variety*, includes three mutually exclusive categories defined by the use of few or many colors. Both the categories about colors were based on Crotti and Magni (2011) and Lüscher et al. (1976). *EMO_Position_horizontal* includes three mutually exclusive categories defined by dividing the surface of the sheet into three equal parts and verifying in which of these parts the largest part of the whole representation is placed: *left, center or right*. Similarly, *EMO_Position_vertical* include the three mutually exclusive categories: *top, center and bottom*. Both the categories on the position were based on Walker and Walker (2007).

##### Motivations: Macro-categories and categories

Motivations refer to reason that the child states on the choice of the represented favorite space and include one macro-category named MOT_Reason, which is defined by 10 non-mutually exclusive categories based on what the child reported about the opportunities offered by space that determine his/her preferences: *Playing* refers to opportunity of playing*, Learning* refers to opportunity of reading, writing, drawing or learning other academic skills*, Observation of nature* refers to opportunity of observing nature in the school environment*, Relationships* refers to opportunity of having relationships*, Privacy* refers to opportunity of having private moments*, Indoor/Outdoor connection* refers to opportunity of passing from indoor spaces to outdoor spaces or *vice-versa, Continuity with family* refers to opportunity of having continuity with family experiences also at school*, Aesthetical reasons* refers to esthetic aspects, such as “it’s coloured,” *Functional reasons* refers to functional aspects of the space, such as “it’s comfortable,” *Others* refers to other reasons not included in the previous ones.

### Analyses

During the coding procedure, each drawing and interview should be coded individually, indicating the frequency of presence of each category. The coding scheme does not necessarily have to be used in its full version: depending on specific interests, only some dimensions, macro-categories or categories may be coded.

In addition to the distribution of frequencies, different analytical approaches can be implemented: individual analyses allow us to investigate the representation of each individual child; group analyses allow us to identify the averages of the aspects emerging from the representation of a group of children, also to investigate differences due to age or gender. Moreover, the analyses can consider each single category, to outline how a group of children represents a specific aspect, or relations among categories, to highlight the patterns of connections that emerge between different categories.

The coding of each drawing and interview should be completed by more than one coder so that inter-rater agreement can be calculated in order to ensure greater validity of the coding procedure.

## Results

To assess the validity of the method, a first application was conducted with a sample of 262 children (141 males, 121 females; mean age = 55.78 months; SD = 11.10; range 37–77 months) from five Italian preschools. The procedure followed the three phases described above in the Procedure section; the only difference was that, due to logistical reasons, the drawings were made in small groups (4/6 children) instead of individually. After a preliminary screening to assess the relevance of the drawings produced, we proceeded with the inter-rater reliability assessment on each category of the coding system, then we evaluated the distribution of each category through frequency analyses, also assessing the relationship between each variable and either age and gender of children through the Chi square test. For the investigation of differences in relation to children’s age, three Age Groups were defined from the distribution in quartiles: Age Group 1 (age less than 25%; mean: 41.96 months); Age Group 2 (aged 25 to 75%; mean: 54.41 months); Age Group 3 (age over 75%; mean: 55.55 months).

### Preliminary screening

From the preliminary screening, 72 drawings were excluded by the researcher as they were not relevant to the task. Thus, scoring and analyses were conducted on a sample of 190 data. A statistically significant relation was found between relevance and age (*p* < 0.001); the relevant drawings were 41.8% in Age Group 1, 79.2% in Age Group 2 and 90.0% in Age Group 3, revealing a developmental trend for relevance.

### Inter-rater reliability (k)

Of the 190 relevant data, a sample of 120 drawings and interviews randomly identified (63%) were evaluated by two independent raters. One of the raters was a Ph.D. student in Psychology (female, 37 years old) who participate in the realization of the coding system; the other one was a masters’ degree student in psychology (male, 28 years old) who was conducting a thesis on children’s drawings and did not participate in the realization of the coding system. Each rater coded each drawing in all the 90 categories. Considering the number of categories of DRAW.IN.G. coding system the 60% of the data was evaluated a reliable sample size for the inter-coders agreement (Sim and Wright, 2005).

Inter-rater reliability was assessed for each macro-category and category by calculating Cohen’s kappa coefficient (k) whose values indicate no agreement (k < 0) or different degrees of agreement, named slight (k range: 0–0.20), fair (k range: 0.21–0.40), moderate (k range: 0.41–0.60), good (k range: 0.61–0.80), excellent (k range: 0.81–1). K values and range for each macro-category are reported in Table 6.

**Table 6:** Categorical inter-rater reliability for each macro-category.

Dimensions	Macro-categories	K value	Range
Physical dimensions			
	PHY_Space	0.629	K 0.60–0.80
	PHY_Specification	0.874	K 0.80–1
	PHY_Location	0.633	K 0.60–0.80
	PHY_Furnishings	0.770	K 0.60–0.80
	PHY_Architecture	0.820	K 0.80–1
Behavioral dimensions			
	BEH_Behavior	0.929	K 0.80–1
Relational dimensions			
	REL_Representation	0.962	K 0.80–1
	REL_Who	0.688	K 0.60–0.80
	REL_Configuration		
	REL_Position_Horizontal	0.714	K 0.60–0.80
	REL_Position_Vertical	0.869	K 0.80–1
Emotional dimensions			
	EMO_Climate	0.689	K 0.60–0.80
	EMO_Archetypes	0.694	K 0.60–0.80
	EMO_Colors_tone	0.610	K 0.60–0.80
	EMO_Colors_variety	0.814	K 0.80–1
	EMO_Position_Horizontal	0.617	K 0.60–0.80
	EMO_Position_Vertical	0.607	K 0.60–0.80
Motivations			
	MOT_Reason	0.694	K 0.60–0.80

### Frequency analyses and Chi square test

The distribution of the categories of each dimension (physical, behavioral, relational, emotional, and motivations) was assessed through frequency analysis; the relation between each variable and either age or gender of children was assessed through Pearson’s Chi square test, considering the frequencies coded from the Ph.D. student, as the rater with more experience in the field. A value of p of <0.05 was considered being statistically significant. *p*-values of the statistically significant relations are reported in the next paragraphs; when they are not reported, it means that there are no statistically significant differences either on gender or age groups in relation to the indicated variable. All percentages and the indication of all *p*-values for each category are reported in Table 7.

**Table 7:** Distribution of frequencies and Chi-square test for Age and Gender for each category of the first administration of the DRAW.IN.G.

Macro-categories	Categories	Frequencies	Frequencies and Chi-square test for age				Frequencies and Chi-square test for gender		
			Ag1	Ag2	Ag3	*p*	M	F	*p*
PHY_Space	Garden	50.0%	60.7%	49.5%	46.0%	0.883	55.2%	44.7%	0.122
	Class	18.9%	10.7%	10.1%	12.7%		8.3%	13.8%	
	Common spaces	18.9%	17.9%	17.2%	22.2%		19.8%	18.1%	
	All the school	10.0%	7.1%	13.1%	6.3%		12.5%	7.4%	
	Eating space	0.5%	0.0%	2.0%	3.2%		0.0%	4.3%	
	Sleeping space	1.6%	0.0%	2.0%	1.6%		0.0%	3.2%	
	Toilet space	0.0%	0.0%	0.0%	0.0%		0.0%	0.0%	
	Other	0.0%	0.0%	1.0%	0.0%		0.0%	1.0%	
PHY_Specificity	Specific	38.9%	32.1%	33.3%	50.8%	0.137	28.1%	50.0%	0.003^**^
	Generic	51.6%	53.6%	58.6%	39.7%		63.5%	39.4%	
	Specific in a general context	6.9%	14.3%	8.1%	9.5%		8.3%	10.6%	
PHY_Location	Indoor	37.9%	28.6%	35.4%	46.0%	0.187	30.2%	45.7%	0.085
	Outdoor	55.3%	67.9%	54.5%	50.8%		62.5%	47.9%	
	Both	6.8%	3.6%	10.1%	3.2%		7.3%	6.4%	
PHY_Architecture^1^	None	68.9%	77.8%	71.4%	61.9%	0.255	68.1%	70.2%	0.752
	Walls	18.4%	33.3%	66.7%	57.7%	0.315	55.2%	63.3%	0.524
	Floors	19.0%	16.7%	59.3%	73.1%	0.077	65.5%	56.7%	0.486
	Ceilings/roof	19.0%	50.0%	63.0%	61.5%	0.839	62.1%	60.0%	0.871
	Doors	5.8%	33.3%	22.2%	11.5%	0.378	17.2%	20.0%	0.786
	Windows	7.9%	50.0%	25.9%	19.2%	0.295	20.7%	30.0%	0.412
PHY_Furnishings^1^	Indoor furnishings	56.3%	16.7%	55.3%	45.0%	0.052	46.7%	43.3%	0.17
	Outdoor furnishings	63.0%	64.3%	48.5%	66.7%	0.697	48.4%	65.6%	0.74
BEH_Behavior	Playing alone	26.8%	28.6%	23.2%	31.7%	0.542	40.6%	23.4%	0.021^*^
	Playing with others	32.1%	35.7%	28.3%	36.5%		17.7%	36.2%	
	Learning moment	4.7%	10.7%	3.0%	4.8%		21.9%	13.8%	
	Observation of nature	8.4%	3.6%	11.1%	6.3%		8.3%	8.5%	
	Privacy moment	1.6%	0.0%	1.0%	3.2%		4.2%	5.3%	
	Not specified	17.9%	17.9%	23.2%	9.5%		5.2%	3.2%	
	Transition or wait	4.2%	0.0%	5.1%	4.8%		1.0%	4.3%	
	Eating moment	2.6%	3.6%	3.0%	1.6%		1.0%	2.1%	
	Sleeping moment	1.6%	0.0%	2.0%	1.6%		0.0%	3.2%	
	Toilet moment	0.0%	0.0%	0.0%	0.0%		0.0%	0.0%	
REL_Representation	People represented	80.0%	57.1%	80.8%	88.9%	0.002^**^	78.1%	81.9%	0.514
	People not represented	20.0%	42.9%	19.2%	11.1%		21.9%	18.1%	
REL_Who^1^	Child him/herself	92.1%	81.3%	90.0%	98.2%	0.051	92.0%	92.2%	0.962
	Friends	44.1%	43.8%	42.5%	46.4%	0.902	48.0%	40.3%	0.337
	Teachers	7.2%	12.5%	10.0%	1.8%	0.132	6.7%	7.8%	0.789
	Familiars	2.6%	6.3%	2.5%	1.8%	0.613	2.7%	2.6%	0.979
REL_Configuration	No one	20%	42.9%	19.2%	11.1%	0.071	21.9%	18.1%	0.854
	Only self	38.4%	37.5%	48.8%	50.0%		44.0%	51.9%	
	Only friends	4.7%	12.5%	7.5%	1.8%		5.3%	6.5%	
	Only teachers	1.0%	0.0%	2.5%	0.0%		1.3%	1.3%	
	Only family members	0.6%	6.3%	0.0%	0.0%		1.3%	0.0%	
	Self&Friends	29.0%	31.3%	31.3%	44.6%		41.3%	31.2%	
	Self&Teachers	3.1%	12.5%	3.8%	0.0%		4.0%	3.9%	
	Self&Family members	1.6%	0.0%	2.5%	1.8%		1.3%	2.6%	
	Self&Friends&Teachers	1.6%	0.0%	3.8%	0.0%		1.3%	2.6%	
REL_Position_horizontal	Left	28.9%	31.3%	25.0%	33.9%	0.756	25.3%	32.5%	0.602
	Center	47.4%	50.0%	51.2%	41.1%		50.7%	44.2%	
	Right	23.7%	18.8%	23.8%	25.0%		24.0%	23.4%	
REL_Position_vertical	Top	2.0%	0.0%	2.5%	1.8%	0.200	2.7%	1.3%	0.712
	Center	51.9%	56.3%	56.3%	28.6%		48.0%	44.2%	
	Bottom	46.1%	43.8%	41.3%	69.6%		49.3%	54.5%	
EMO_Climate	Positive	74.6%	71.4%	67.6%	84.9%	0.326	62.9%	86.8%	0.015^*^
	Negative	0.7%	0.0%	1.4%	0.0%		17.1%	5.9%	
	Mixed	1.5%	0.0%	2.8%	3.8%		1.4%	0.0%	
	Neutral	11.6%	7.1%	16.9%	5.7%		5.7%	0.0%	
	Not represented	10.1%	21.4%	11.3%	5.7%		12.9%	7.4%	
EMO_Archetypes^1^	Land line	80.0%	88.9%	73.3%	86.5%	0.171	81.0%	78.8%	0.779
	Skyline	64.3%	55.6%	71.7%	56.8%	0.230	65.1%	63.5%	0.857
	Sun	66.1%	58.8%	63.3%	75.7%	0.534	63.5%	70.6%	0.459
	Moon	0.0%	0.0%	0.0%	0.0%	-	0.0%	0.0%	-
	Trees	39.1%	55.6%	28.3%	48.6%	0.071	39.7%	38.5%	0.894
	Flowers	17.4%	11.1%	21.7%	13.5%	0.439	14.3%	21.2%	0.333
	Rainbow	4.0%	0.0%	8.3%	0.0%	0.091	6.3%	1.9%	0.247
	Animals	10.4%	16.7%	8.3%	10.8%	0.595	7.9%	13.5%	0.335
	Monsters	0.0%	0.0%	0.0%	0.0%	-	0.0%	0.0%	-
EMO_Colors_tone	Warm colors	11.1%	7.1%	16.2%	17.5%	0.241	13.5%	17.0%	0.001^***^
	Cold colors	19.4%	35.7%	28.3%	20.6%		39.6%	13.8%	
	Both	42.5%	57.1%	55.6%	61.9%		46.9%	69.1%	
EMO_Colors_variety	One color	6.5%	7.1%	9.1%	9.5%	0.227	11.5%	6.4%	0.026^*^
	Up to four colors	22.9%	39.3%	35.4%	22.2%		38.5%	24.5%	
	More than four colors	43.1%	53.6%	55.6%	68.3%		50.0%	69.1%	
EMO_Position_horizontal	Left	8.9%	0.0%	8.1%	14.3%	0.087	7.3%	10.6%	0.870
	Center	88.4%	100.0%	87.9%	84.1%		89.6%	87.2%	
	Right	2.6%	0.0%	4.0%	1.6%		3.1%	2.1%	
EMO_Position_vertical	Top	2.1%	0.0%	3.0%	1.6%	0.483	2.1%	2.1%	0.771
	Center	84.2%	92.9%	80.8%	79.4%		84.4%	79.8%	
	Bottom	15.8%	7.1%	16.2%	19.0%		13.5%	18.1%	
MOT_Reason^1^	Playing	75.6%	18.1%	50.3%	31.6%	0.197	52.5%	47.5%	0.808
	Learning	5.6%	38.5%	23.1%	38.5%	0.104	53.8%	46.2%	0.949
	Observation of nature	11.5%	33.3%	48.1%	18.5%	0.156	45.7%	54.3%	0.266
	Relationships	19.7%	19.6%	39.1%	41.3%	0.129	51.9%	48.1%	0.900
	Privacy	4.7%	9.1%	54.5%	36.4%	0.617	54.5%	45.5%	0.916
	Indoor/outdoor connection	1.7%	0.0%	75.0%	25.0%	0.504	50.0%	50.0%	0.904
	Continuity with family	2.6%	16.7%	66.7%	16.7%	0.694	83.3%	16.7%	0.131
	esthetic reasons	1.3%	33.3%	33.3%	33.3%	0.808	33.3%	66.7%	0.492
	Functional reasons	2.1%	8.3%	50.0%	41.7%	0.460	66.7%	33.3%	0.330
	Others	10.3%	29.2%	45.8%	25.0%	0.535	41.7%	58.3%	0.241

1 Macro-category including non-mutually exclusive categories.

Ag1, Age Group 1; Ag2, Age Group 2; Ag3, Age Group 3; M, Male; F, Female; *p*, *p*-value from Chi-Square analyses.

* *p* ≤ 0.05;

** *p* ≤ 0.01;

*** *p* ≤ 0.001.

#### Physical dimension

As for *PHY_Space*, the distribution of frequencies indicates that half of the children represented the outdoors (50.0%); the class and the common spaces are the second favorite spaces, represented by the same percentage of children (18.9%). Some of children made a general representation of all the school (10.0%) and a few children represented the sleeping room (1.6%) or the eating room (0.5%).

As for *PHY_Specificity*, the distribution of frequencies indicates that most children (51.6%) represented generic space, 38.9% of the children represented specific space and 6.9% of the children represented a specific space also drawing a general context. The Chi square test indicates statistically significant differences between males and females for *PHY_Specificity* (*p* = 0.003): the majority of males (63.5%) represented more generic space while the majority of females (39.4%) represented specific spaces.

As for *PHY_Location*, the distribution of frequencies indicates that most children represented outdoor spaces (55.3%), 37.9% represented indoor spaces and 6.8% represented both outdoor and indoor spaces. As for *PHY_Architecture*, the distribution of frequencies indicates that only 31.1% of children represented such elements, while 68.9% did not represent them. As for *PHY_Furnishings*, indicates that most children represented indoor (56.3%) or outdoor furnishings (63%).

#### Behavioral dimension

As for *BEH_Behavior*, most of children represented playing with others (32.1%) or playing alone (26.8%). 17.9% of children did not specify the behavior represented. 8.4% of the children represented the observation of nature, 4.7% moments of learning, 4.2% moments of transitions or waits, 2.6% eating moments, 1.6% moments of privacy and 1.6% sleeping moments. The Chi square test indicates statistically significant differences between males and females for *BEH_Behavior* (*p* = 0.021): the majority of males (40.6%) represented playing with others while the majority of females (36.2%) represented playing alone. The second most represented behavior was an unspecified activity for males (21.9%) and playing with others for females (23.4%). The third most represented situation was playing alone for males (17.7%) and an unspecified activity for females (13.8%).

#### Relational dimension

As for *REL_Representation*, most children (80%) represented at least one person in their drawings, while 20% of children did not represent people. The Chi square test indicates statistically significant differences between age groups for *REL_Representation* (*p* = 0.002): at least one person was represented in 57.1% of children belonging to Age Group 1, in 80.8% belonging to Age Group 2 and in 88.9% of children belonging to Age Group 3, revealing a developmental trend for the representation of people. As for *REL_Who*, the distribution of frequencies indicates that, of the children representing people, the great majority (92.1%) represented themselves, 44% represented friends, 7.2% represented teachers and 2.6% represented family members. As for *REL_Configuration*, the distribution of frequencies indicates that most children represented only themselves (38.4%) or themselves with friends (29.0%). Some children represented only friends (4.7%), only teachers (1.0%) only family members (0.6%), themselves with teachers (3.1%), themselves with familiars (1.6%) or themselves with both friends and teachers (1.6%).

As for *REL_Position_horizontal*, most of children (47.4%) represented people in the middle of the drawing, 28.9% to the left and 23.7 to the right of the drawing. As for *REL_Position_vertical*, most children (51.9%) represented people in the middle of the drawing, 2% to the top and 46.1% to the bottom of the drawing.

#### Emotional dimension

As for *EMO_Climate*, of the children representing people, the majority (74.6%) represented a positive emotional climate, 11.6% represented a neutral emotional climate, 1.5% represented mixed emotional climate and only one child (0.7%) represented a negative emotional climate. 10.1% of children did not represent the emotional climate. The Chi square test indicates statistically significant differences between males and females for *EMO_Climate* (*p* = 0.015): positive emotional climate was represented more by females (86.8%) than males (62.9%), while neutral emotional climate was more represented by males (17.1%) than females (5.9%). The non-representation of emotional climate also was more frequent in males (12.9%) than females (7.4%). Finally, males were the only ones who represented a mixed (1.4%) and negative (5.7%) emotional climate. As for *EMO_Archetypes*, the most depicted were the land line (80.0%), the skyline (64.3%) and the sun (66.1%), followed by trees (39.1%), flowers (17.4%), animals (10.4%) and a rainbow (4%). No children depicted the moon or monsters.

As for *EMO_Colors_tone*, most children (42.5%) used both warm and cold colors in their drawings, while 19.4% used cold colors and 11.1% used warm colors. The Chi-square test indicates a statistically non-significant relation between such variable and age, while it indicates statistically significant differences between males and females for *EMO_Colors_tone* (*p* = 0.001): both colors were used by 46.9% of males and 69.1% of females. Males used more cold (39.6%) than warm colors (13.5%), while females used more warm (17.0%) than cold (13.8%) colors. As for *EMO_Colors_variety*, most children (43.1%) used many colors in their drawings, while 22.9% used up to four colors and 6.5% used only one color. The Chi square test indicates statistically significant differences between males and females for *EMO_Colors_variety* (*p* = 0.026): more than four colors were used by 50% of males and 69.1% of females. More than four colors were used by 38.5% of males and 24.5% of females. One color was used by 11.5% of males and 6.4% of females. As for *EMO_Position_horizontal*, most children (88.4%) drew in the middle of the sheet, while 8.9% drew to the left and 2.6% to the right of the sheet. As for *EMO_Position_vertical*, most children (84.2%) drew in the center of the sheet, while 15.8% drew at the bottom and 2.1% at the top of the sheet.

#### Motivation

As for *MOT_Motivation*, most of the cited motivations (75.6%) referred to playing opportunities, 19.7% to relationships, 11.5% to the observation of nature, 5.6% to learning opportunities, 5.1% to functional aspects, 4.7% to opportunities for privacy, 2.5% to the continuity between school and family, 1.7% to the continuity between indoor and outdoor spaces, and 1.3% to esthetic aspects.

## Discussion

The study indicated the general appropriateness of the method, in addition to its feasibility, also revealing some critical aspects to consider. First, the method is easy as it takes little time for its administration, i.e., about half an hour for drawing and about 5 min for each interview. Second, it is ecological, as it engages children using tools familiar to them. Third, it is multifaceted, as it analyses different aspects of the representation of space, allowing us to grasp a complex vision of children’s experience of their ECEC environment. Fourth, it is flexible, as it allows easy adaptations depending on specific situations and interests, choosing some specific aspects of the space to be investigated.

Regarding the reliability of the instrument, it should be noted that the inter-rater indices revealed a good to excellent agreement for all identified categories, with the exception of only two categories within *PHY_Architecture*: *chimney* and *fence*. This can be due to the fact that such categories were taken from the House Drawing Task but chimneys and fences are not in fact significant elements in the representation of the school building, so probably children did not depict them for this reason. The good to excellent inter-rater agreement of all the other categories indicates that they are clearly defined and allow for consistent assessments, indicating their appropriateness in the analysis of ECEC space representation. The high agreement could also allow for the use of the DRAW.IN.G. method, not only by researchers, but also by professionals who work with children in the educational field, such as teachers and pedagogical coordinators, after a training course in the use of the tool.

The distribution of the categories shows that there are no substantial differences in relation to either the different age groups or gender, except for some aspects already consolidated in the literature. This highlights, in general, how this method is suitable in preschool age regardless gender and is not particularly influenced by the age of children. However, with regard to the age factor, it should be noted that the validation study, as we have seen, shows that only 41.8% of the drawings of children in the lower age group appear to be relevant to the task. These data may indicate the age of 3 years as the age limit of use and that it is therefore preferable to use the DRAW.IN.G. tool with children with children aged 4 and over.

Among the other differences emerging for either age or gender, significant relations were found between age and *REL_Representation* and between gender and the following macro-categories*: PHY_Specificity*, *BEH_Behavior, EMO_Climate, EMO_Colors_tone* and *EMO_Colors_variety.*

As for *REL_Representation*, a significant developmental trend was observed as younger children represented fewer people than older ones, in line with classical studies that argue that social sensitivity increases with age (Piaget, 1926; Mossler et al., 1976). As for *PHY_Specificity* and *BEH_Behavior*, it is interesting to note that males represented more generic spaces and situations where they play with others, while females represented more specific spaces and situations where they play alone. These findings are in line with classic literature indicating that girls are usually engaged in more intimate play and smaller groups, compared to boys (Lever, 1998) and that girls are more oriented in small group interactions, whereas boys tend to choose more physical activities (Maccoby, 1990).

As for the *EMO_Climate* it was found that positive emotional climate was represented more by females, while neutral emotional climate was more represented by males; furthermore, the rare representation of mixed and negative emotional climate was found only in drawings provided by males. Consistently, in relation to the use of colors, cold colors were used more by males while warm colors were used more by females (*EMO_Colors_tone*), and the variety of colors was found to be higher in females than males (*EMO_Colors_variety*). Such findings are in line with existing literature showing gender differences in children’s emotional expression, with females showing more positive and internalizing emotions than males, and males showing more externalizing emotions (e.g., anger) than girls (Chaplin and Aldao, 2013).

The specific distribution of frequencies for each variable in relation to the existing literature will not be discussed here, as it is not the core of the present article; it is important here to note that there is a great variability in the frequency distribution of the different categories, indicating that they seem able to discriminate and bring out the different aspects of children’s representation of their ECEC environment and the experience they have with it. Furthermore, it is interesting to note that some categories (e.g., *Outdoors* within *PHY_Space*; *Playing with others* or *Playing alone* within *BEH_Behavior*) are particularly recurrent in the representations of children, while others are less frequent but equally interesting and worthy of attention (e.g., *Sleeping room* within *PHY_Space*; *Privacy moments* or *transitions or waits* within *BEH_Behavior*). A discussion on such contents should be found in Berti et al., 2022.

Concerning the possible application of DRAW.IN.G. as a methodological tool, some considerations should be made in relation to the fact that the effort of developing such a tool would be useful and important both in the field of research and practice.

As for the research, the standardization of a tool that allows us to explore children’s meanings about their ECEC spaces covers some literature gaps related to the need to develop systematic methods to use drawing in research with children, the need to integrate drawings and interviews and the need to explore the use of drawings for the investigation of children’s meanings on specific topics. Furthermore, the construction of the coding system, based on both bottom-up and top-down processes, enriches the tool, including evaluation parameters both built *ad hoc* and already existing in the literature. An added value of the tool is that in fact it consists of both “descriptive” and “projective” aspects for the evaluation of the contents of the drawings. Although the scientific value of projective tools has been questioned in psychology for some time, we think that an integration of both the mentioned aspects could provide a complex and articulated vision about children’s experience of the school environment and reflect on different aspects related to it. Furthermore, another strength of this method is that it can be replicated even on the same group of children. For example, if significant spatial changes are made in an educational context, it could be of fundamental importance for professionals working in this context to understand whether and how such changes have an impact on the experiences and representations of children. In this case, the DRAW.IN.G. tool could be used before and after the changes, eventually adapting a specific task to the specific aim. Another important potential of the method is that, in addition to the distribution of the categories, which highlights the relevance of different aspects of children’s representation of space, the tool allows us to identify some specific configurations of different dimensions of children’s experience of their ECEC environment. This aspect is very interesting for research, as it reveals complex and mostly unconscious relations among the different investigated dimensions that such young children might not be able to explain verbally.

As for the practice, the tool might have a great relevance in the spatial design processes of the ECEC centers; for example, it could be very interesting for a teacher or a coordinator to understand what kind of experience and representation children have of the educational space. In fact, very often the space is thought of by adults, and children are seen as “users” of this space. Nevertheless, recent studies in this area indicate that the involvement of children in design issues represents a way that fosters their development and well-being (Nah and Lee, 2016; Botsoglou et al., 2017). In this sense DRAW.IN.G. is a tool to make children’s point of view on the educational environment more accessible to teachers. In addition, both the drawing and the interview, intended as a narration/conversation by the children, are methodologies widely used in educational contexts and are therefore quite familiar to teachers.

For this purpose, simplified variants of the coding grid could be realized. In particular, an adaptation for teachers could include the elimination of some more specific categories, such as the use of archetypes or the position of the drawing in the sheet, while it could focus on some more significant categories from an educational point of view, such as the place represented, the preference for indoor or outdoor spaces, or the inclusion of relationships in the ECEC environment. Adapted versions should focus on specific aspects (e.g., children’s perceptions of specific spaces) not including other potentials of the tool more related to research issues (e.g., relations between categories).

Despite the relevance of the standardization of the DRAW.IN.G. tool presented in this article, it is necessary to highlight some methodological limitations. A first important critical aspect emerged from the preliminary analysis of the drawings: as expected, a significant developmental trend on relevance was found. Such a trend indicates that most children aged 3 years had difficulty understanding the task, in fact over 58% of them produced an irrelevant drawing. This datum is in line with classic literature which indicates that as children grow up, the better they are at understanding a drawing task and providing an appropriate response (Luquet, 1913; Piaget, 1929). The finding indicates that the DRAW.IN.G. tool could be more appropriate for children aged 4 years or more; the percentage of not relevant drawings in children aged 4/5 years (about 20%) and 5/6 years (about 10%) is, in fact, acceptable. As it has emerged that not all preschool children understand the task, variants or simplifications could be imagined to investigate even the point of view of the youngest. Further studies involving larger and more heterogeneous samples could clarify this aspect. Future research could also verify the adaptation of this method also for older school age children, such as the ones aged 6 or 7.

Second, although the procedure requires drawings to be made individually, logistical requirements may determine the need to make drawings in small groups of children. Such requirements could, for example, relate to the availability of markers or the arrangement of tables in the classroom, as was the case for the preliminary study. The preliminary study showed that the realization of the drawings in small groups (4–6 children) is feasible; this condition may also be considered favorable for the ecological viability of the administration, when it represents the usual way in which children are used to drawing in the classroom. However, the risk of imitation and copying between children should be considered among limitation, as it could affect the frequency analyses on the elements represented.

Third, in relation to projective indicators included in the coding grid, such as the representation of archetypes or the use of colors, there may be a risk of overestimation of the meanings of the elements that children usually represent in their drawing (e.g., the sun) or possible distortions due to different social habits in males and females related to the use of colors. However, we believe that the inclusion of projective indicators in the DRAW.IN.G. tool could be an added value, especially for the investigation of the emotional dimension, in addition to the facial expression depicted in the drawings: since literature indicates children’s preference for the representation of happy expression in the early age (Cannoni et al., 2021), we believe it is important to include other indicators to detect the emotional tonality of children’s experience.

Fourth, also in relation to the possibility of subjective interpretations for projective indicators, it would also be necessary to verify the concurrent validity of DRAW.IN.G. At this stage, such verification is not possible, because to test the concurrent validity of an instrument it is necessary that the data obtained with this tool are compared with those obtained by another different and validated tool that measures the same constructs or similar constructs that are supposed to be related (parameters). To our knowledge, there is no validated tool that measures children’s experience representation of their school space, and it is also difficult to identify an external criterion/parameter since there are still few empirical studies concerning the investigated construct and therefore a scarce literature on the topic. When this aspect will be more studied, further studies should hypothesize related external parameters to evaluate the concurrent validity. Concerning this issue, we argue that the inclusion in the tool of an interview, in addition to the drawing, can represent a sort of control with respect to the information collected through the drawing, as widely supported by different authors (Yuen, 2004; Darbyshire et al., 2005; Bland, 2012).

Finally, it would also be interesting to validate this tool with children of other nationalities, in addition to the Italian one, to verify whether any differences related to the organization of educational services, and the educational value that is attributed to the spaces themselves, could affect the perceptions of children.

Beyond the aforementioned limits, DRAW.IN.G. represents an attempt to advance our knowledge in the field of the use of drawing as a research tool, trying to bridge an important methodological literature gap. The method presented should be used both in research and in practice revealing interesting potential to bring us closer to children’s point of view on their perception of ECEC environment.

## Data availability statement

The original contributions presented in the study are included in the article/Supplementary material, further inquiries can be directed to the corresponding author.

## Ethics statement

Ethical review and approval was not required for the study on human participants in accordance with the local legislation and institutional requirements. Written informed consent to participate in this study was provided by the participants’ legal guardian/next of kin.

## Author contributions

SB and AC designed the study and analyzed the results. SB implemented the first application of the method under the supervision of AC. SB wrote the manuscript. AC revised the manuscript. All authors contributed to the article and approved the submitted version.

## Conflict of interest

The authors declare that the research was conducted in the absence of any commercial or financial relationships that could be construed as a potential conflict of interest.

## Publisher’s note

All claims expressed in this article are solely those of the authors and do not necessarily represent those of their affiliated organizations, or those of the publisher, the editors and the reviewers. Any product that may be evaluated in this article, or claim that may be made by its manufacturer, is not guaranteed or endorsed by the publisher.
