# Supplementary material for: DRAW.IN.G.: A tool to explore children’s representation of the preschool environment
Source: Front Psychol. 2022 Dec 20;13:1051406. doi: 10.3389/fpsyg.2022.1051406 (PMC9807652; doi:10.3389/fpsyg.2022.1051406)
Supplement: Supplementary file 1 [file Data_Sheet_1.pdf]

Appendix

Preliminary Phase: contents and examples of quotes for researcher's presentation

Content	*Example of quote*
Presentation of the researcher	*Hi kids, I'm John and I'm a researcher, in other words, an inquisitive person trying to understand a few things"*
Presentation of the research	*"Today I would like to understand which are the favorite places of children in their schools, and when I understand it I can write a book so others can find out about my discovery!"*
Presentation of the task	*"Your school is made up of many places, some indoor others outdoor, so I will ask you to make a drawing on the place that you like the most here at school. You can draw any place you like, and you can also draw yourself or your teachers or other children or other people, as you like. When you have finished the drawing, I will ask you what you have drawn, so I can understand better"*
Asking for informal consent	*"Would you make a drawing for my book, so I can figure out what your favorite place is here at school?"*

Drawing phase: description of the main characteristics

Characteristics	*Description*
Task	*"Please draw the place where you like to stay the most when you are here at school"*
Materials	A white sheet of A4 paper for each child and markers of various colors (red, yellow, blue, orange, green, violet, pink, brown, gray, black) for each child or for each small group of children
Time	All the time needed (usually the activity takes about half an hour)

Interview phase: description of the main characteristics

Characteristics	*Description*
Task	Questions of the Interview Grid [Figure 1]
Materials	Interview grid and a pen to note the child's answers, a pencil to indicate the elements on the drawing, a digital tool to audio-video record the interview
Time	All the time needed (usually the activity takes about 5 minutes)
