# Supplementary material for: DRAW.IN.G.: A tool to explore children’s representation of the preschool environment
Source: Front Psychol. 2022 Dec 20;13:1051406. doi: 10.3389/fpsyg.2022.1051406 (PMC9807652; doi:10.3389/fpsyg.2022.1051406)
Supplement: Supplementary file 2 [file Data_Sheet_2.PDF]

Supplementary Figures (SF) | Example of drawings for each micro-category and for not relevant drawings

1. **PHY_Space**

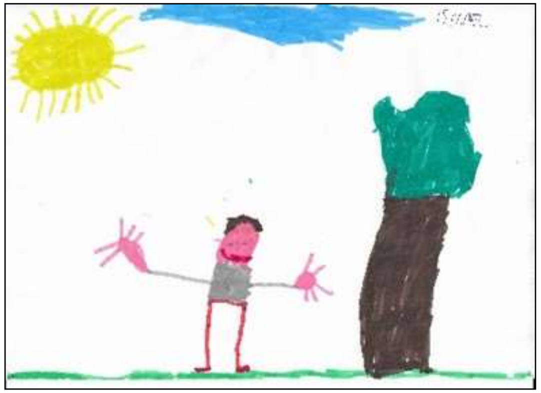

SF 1.1 | Outdoors: *"That's me in the garden"*

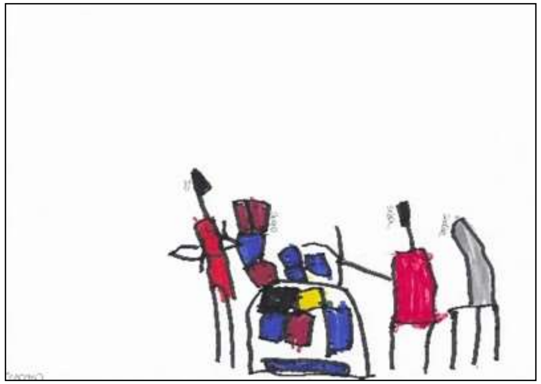

SF 1.2 | Class: *"These are me and my friend Seba playing with Lego bricks on the table with a chair in the class"*

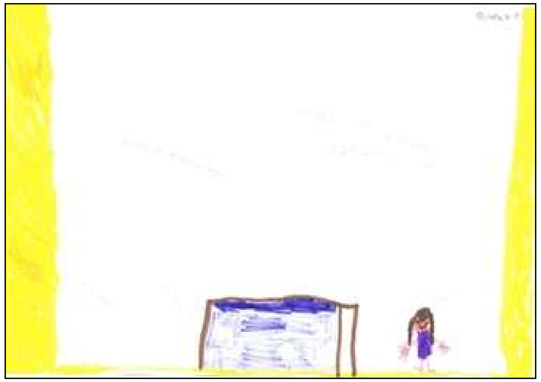

SF 1.3 | Common spaces: *"This is me in the corridor looking at myself in the mirror"*

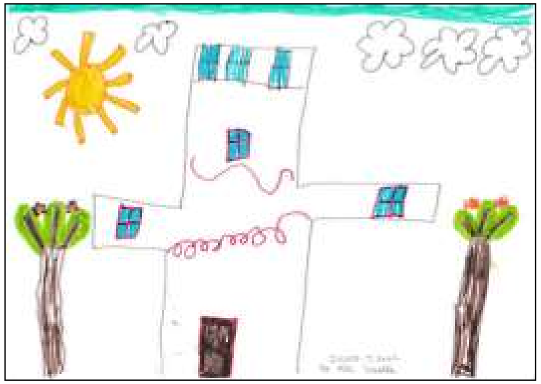

SF 1.4 | All the school: *" I drew the whole school"*

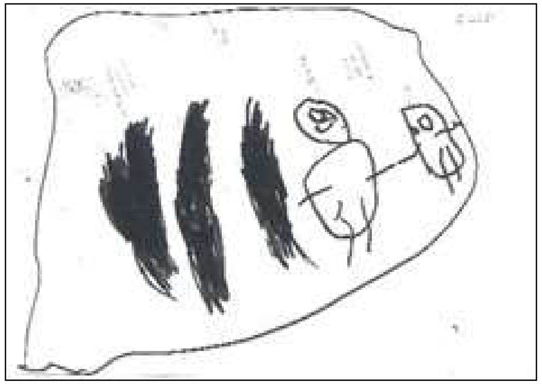

SF 1.5 | Eating space: *"These are me and my nanny, we're going to eat on the tables"*

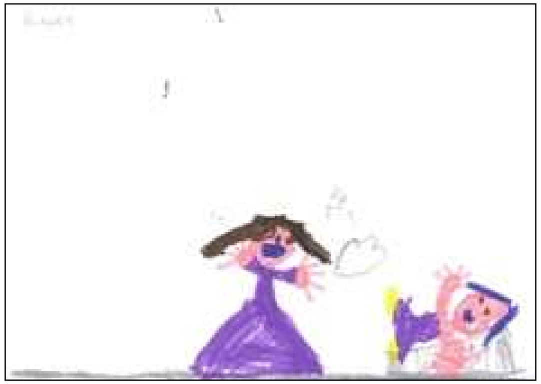

SF 1.6 | Sleeping space: *"This is me in the sleeping room cuddling a girl younger than me"*

2. **PHY_Specificity**

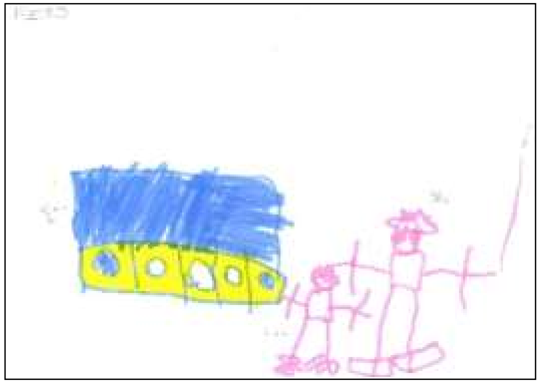

SF 2.1 | Specific: *" I drew the bench: it contains small plastic animals that I like to play with"*

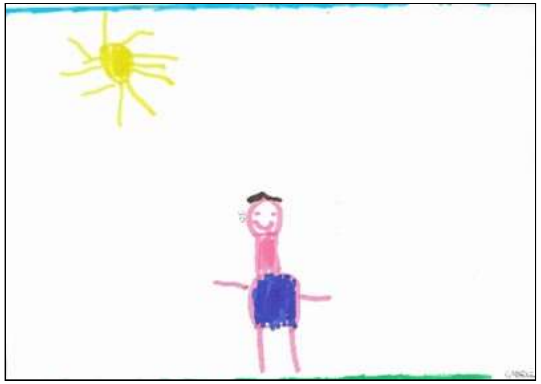

SF 2.2 | Generic: *"Me in the school garden"*

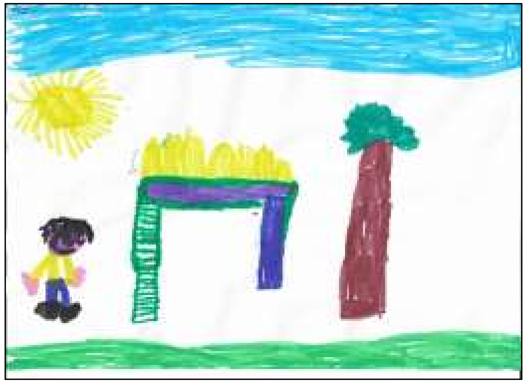

SF 2.3 | Specific in a general context: *"I'm in the garden playing with slides "*

3. **PHY_Location**

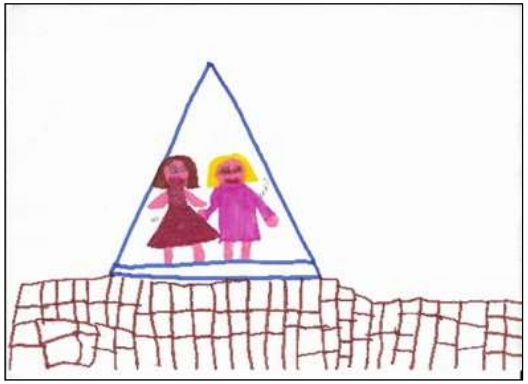

SF 3.1 | Indoor: *"Me and my friend Emma inside the pyramid in the corridor, and the floor"*

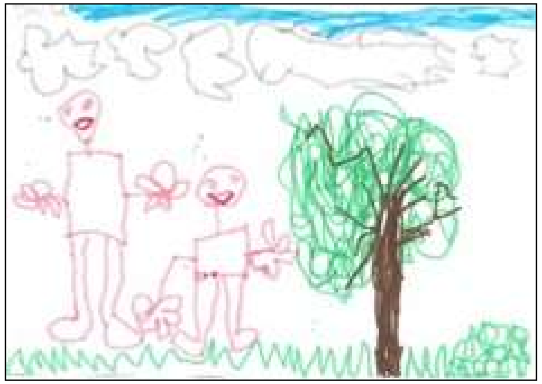

SF 3.2 | Outdoor: *"Me and my friend Giovanni in the school garden"*

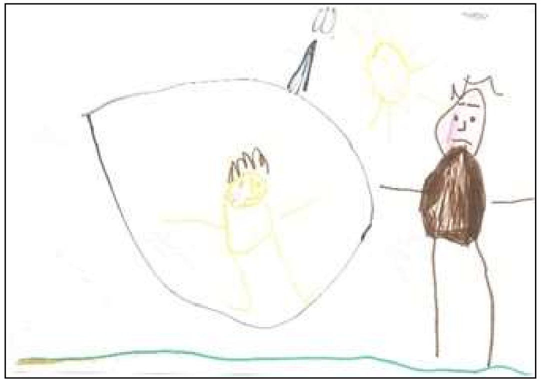

SF 3.3 | Both: *"This is me inside the school while I'm wearing to go out, and this is my friend Jacopo waiting for me in the garden"*

4. **PHY_Furnishings**

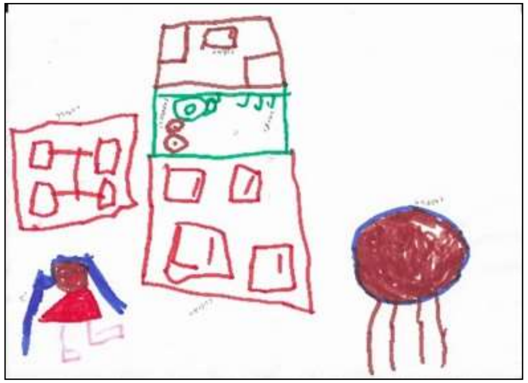

SF 4.1 | Indoor furnishings: *"This is me in the class playing with the kitchen: there are vegetables and dishes, the table, the hooks for hanging pots, the place to put the bottles and the oven"*

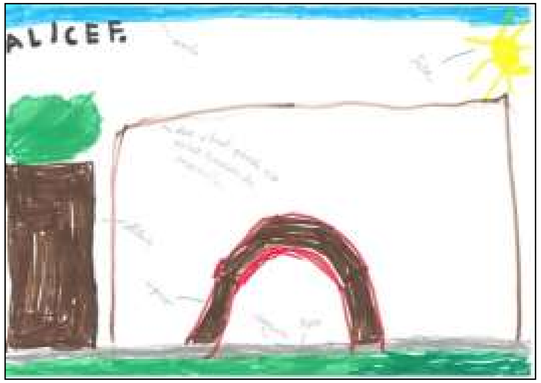

SF 4.2 | Outdoor furnishings: *"This is the gazebo we have in the garden, where we have a snack in the afternoon"*

5. **PHY_Architecture**

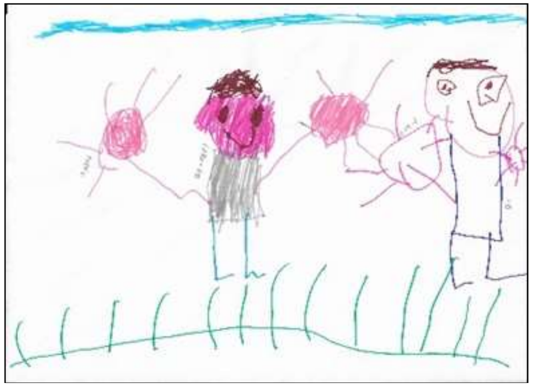

SF 5.1 | None: *"Me and my friend Lorenzo on the grass in the garden"*

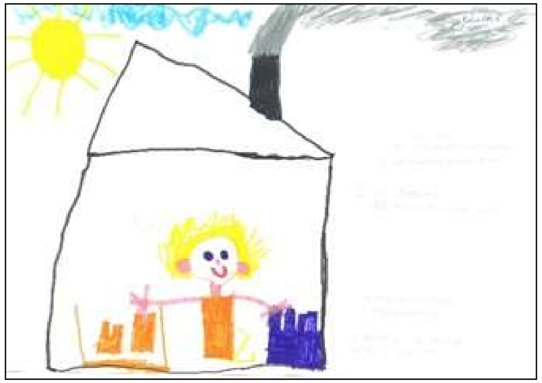

SF 5.2 | Walls, Floors, Ceilings: *"This is me playing with Lego bricks in the school"*

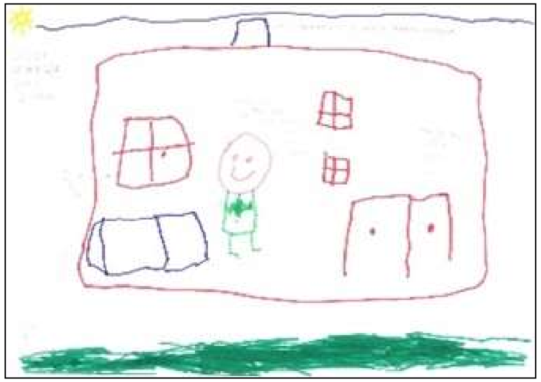

SF 5.3 | Doors, Windows: *"This is me in the school with the door and the windows. And this blue one is my bed"*

6. **BEH_Behavior**

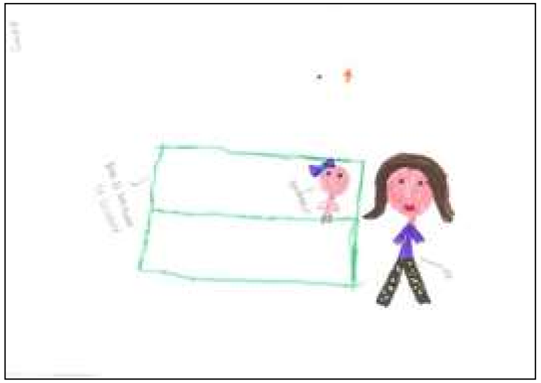

SF 6.1 | Playing alone: *"I drew that I'm playing with dolls and the two closets containing dolls"*

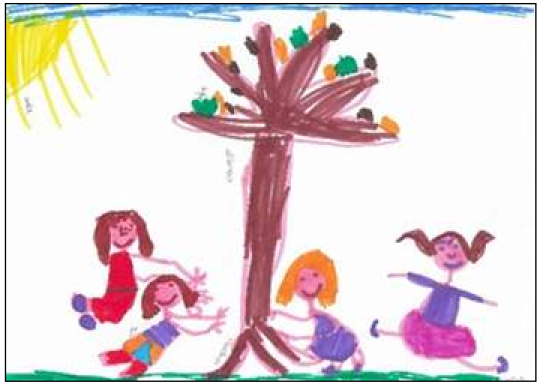

SF 6.2 | Playing with others: *"These are me and my friends playing under the tree: we're buliding a nest with leaves"*

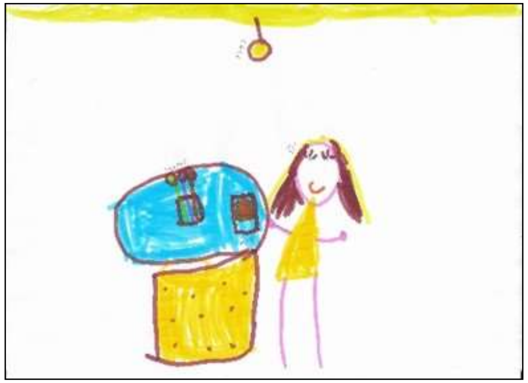

SF 6.3 | Moments of Learning: *"This is me near the table in my class; I'm writing on a paper with the pencils"*

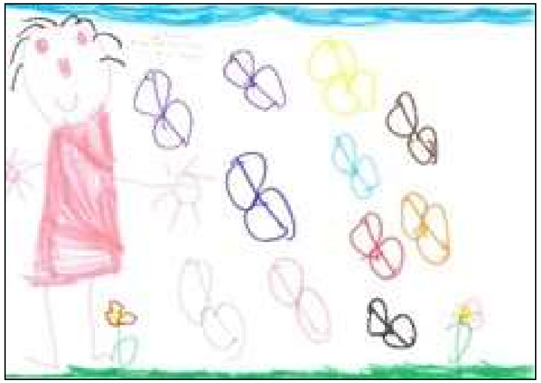

SF 6.4 | Observation of nature: *"I drew me in the school garden looking at the butterflies"*

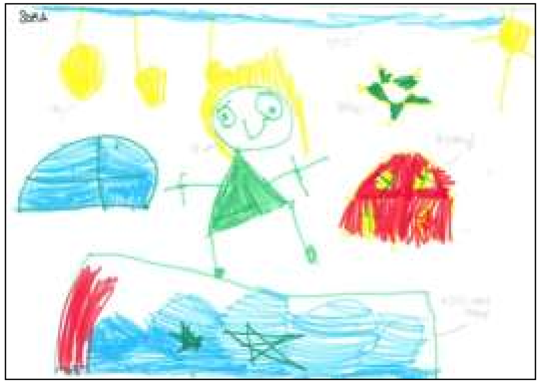

SF 6.5 | Moments of privacy: *"I drew the relax room where I can take refuge when there is too much confusion in the hall because the boys run"*

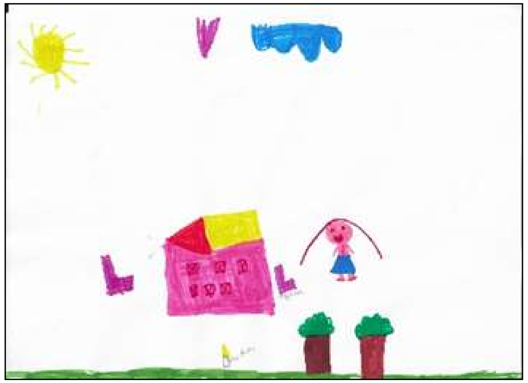

SF 6.6 | Moments of transition or wait: *"This is me arriving at school"*

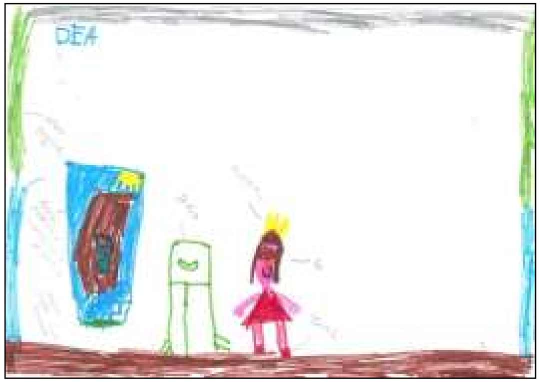

SF 6.7 | Eating moment: *“I drew the class when I did the waitress for lunch"*

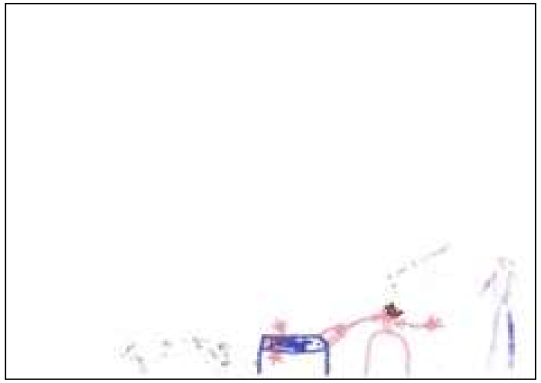

SF 6.8 | Sleeping moment: *“This is me in the sleeping room while I’m cuddling a girl that is younger than me”*

7. **REL_Representation**

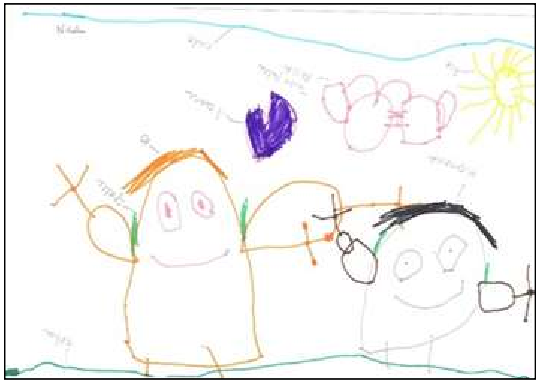

SF 7.1 | People represented: *" I drew me and my friend in the school garden"*

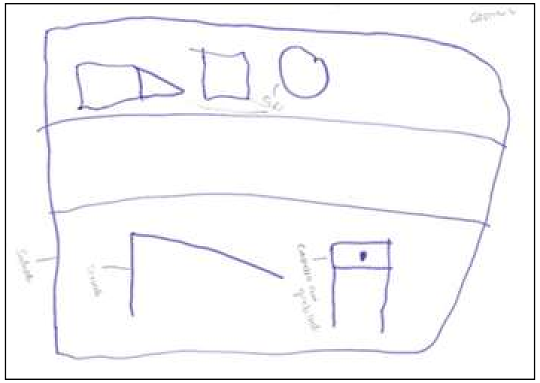

SF 7.2 | People not represented: *"This is the hall with a drawer with toys and dome cubes"*

8. **REL_Who**

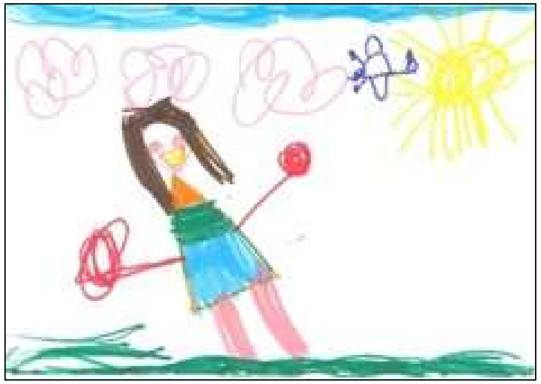

SF 8.1 | Child him/herself: *" I drew me in the school garden with the sun, the clouds and a bird"*

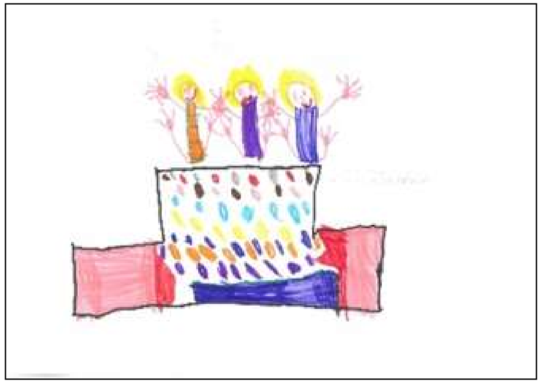

SF 8.2 | Friends: *"These are my friends Alberto and Chiara and me playing with the tower"*

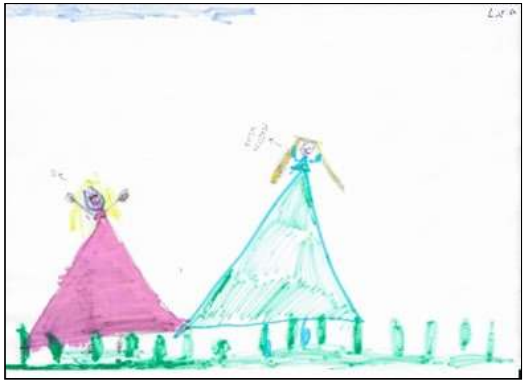

SF 8.3 | Teachers: *"This is me with my teacher in the school garden"*

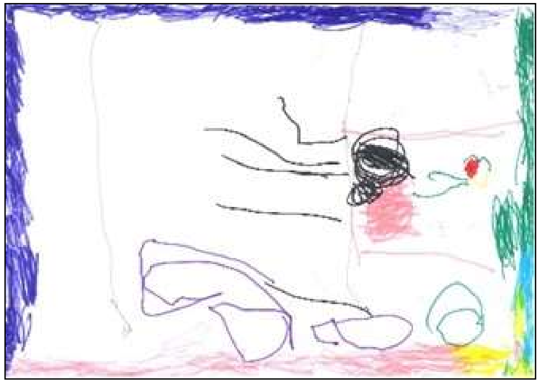

SF 8.4 | Familiars: *“This is me with my mum in the school garden"*

9. **REL_Configuration**

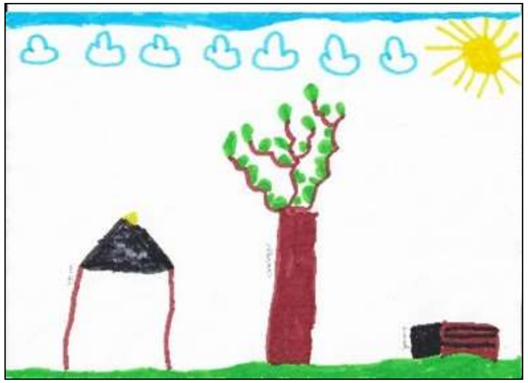

SF 9.1 | No one: *“I drew the school garden with the little wooden house and the tunnel and a tree”*

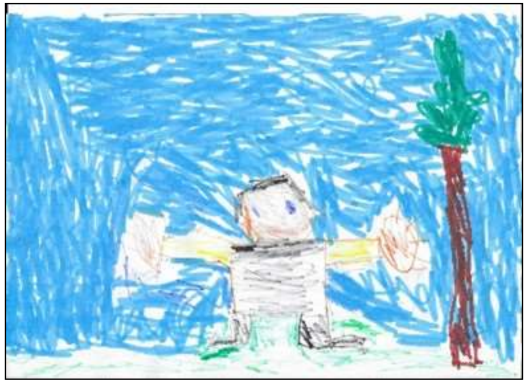

SF 9.2 | Only self: *“I drew me in the school garden with a tree and the sky”*

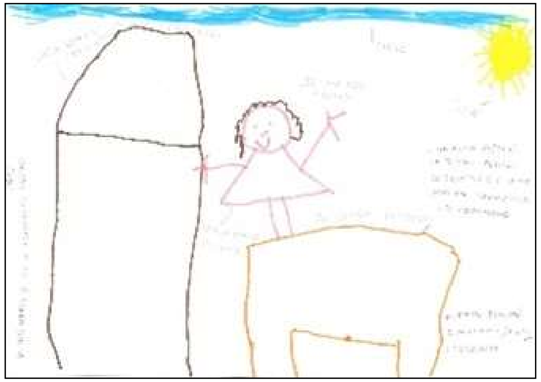

SF 9.3 | Only friends: *“This is my friend Serena: she’s on the bench near the wooden house that is in the garden”*

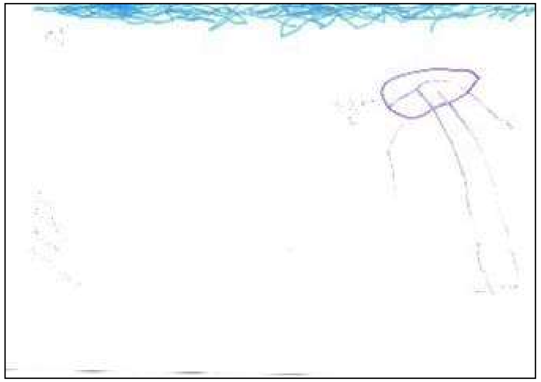

SF 9.4 | Only teachers:*"This is nanny Valeria in the school garden with the sky"*

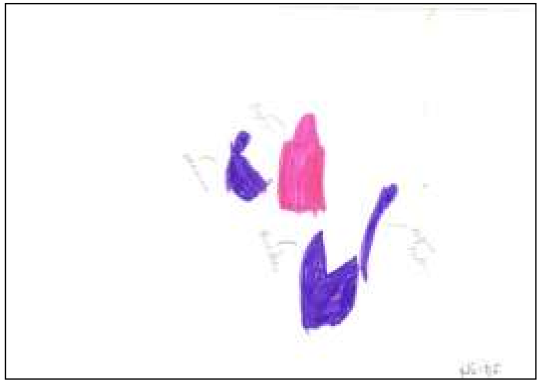

SF 9.5 | Only familiars: *"The blu ones are my brother, my mum and my dad, and the pink one is the kitchen of the school"*

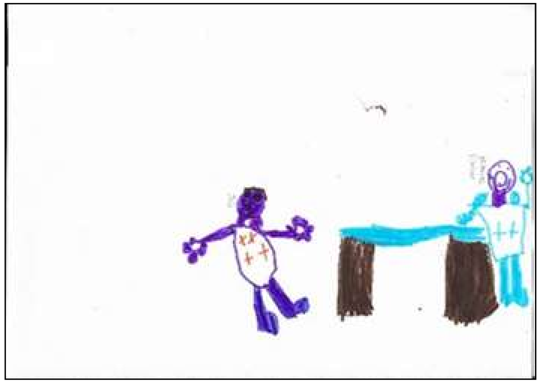

SF 9.6 | Self & Friends: *"This is me with my friend Pietro playing Lego in the class"*

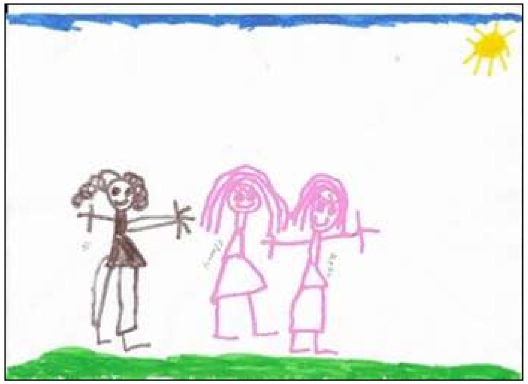

SF 9.7 | Self & teachers:*" I drew me, nanny Franci e nanny Dany"*

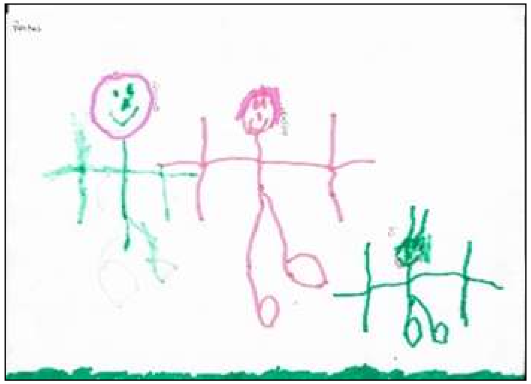

SF 9.8 | Self & familiars: *"That's me, my mum and my grandpa in the school garden"*

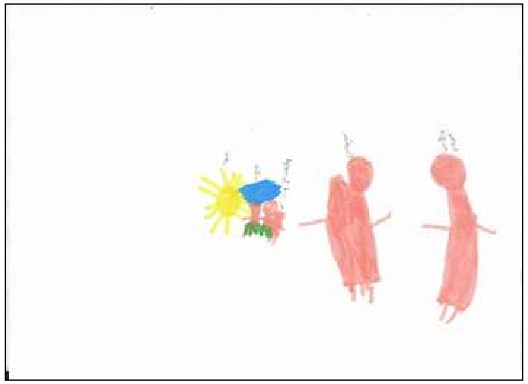

SF 9.9 | Self&Friends&Teachers: *"The garden with the children, there are Cecilia, Giuseppe and I, the sky, the sun, nanny Paola and nanny Angela"*

10. REL_ Position_Horizontal

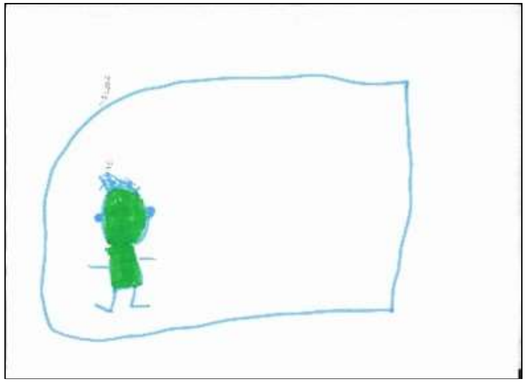

SF 10.1 | Left: *" This is me in the hall watching a movie"*

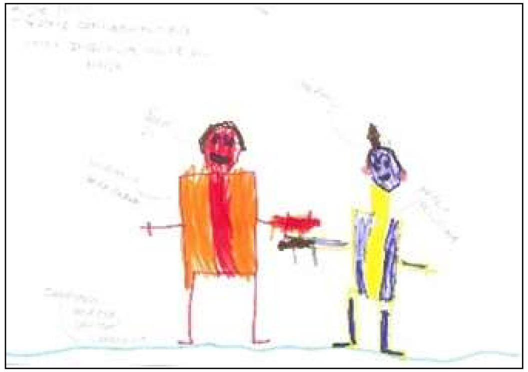

SF 10.2 | Center: *"These are me and my friend Gherri in the corridor with the t-shirts of our favorite football teams"*

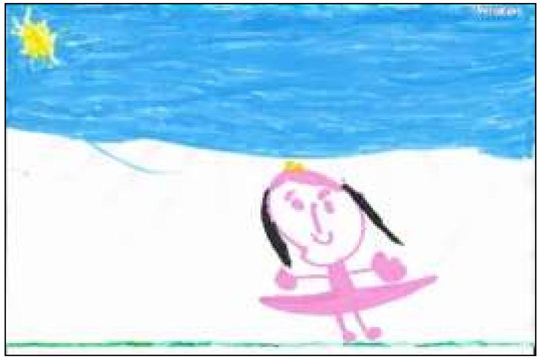

SF 10.3 | Right: "*I drew me running in the school garden"*

11. REL_ Position_Vertical

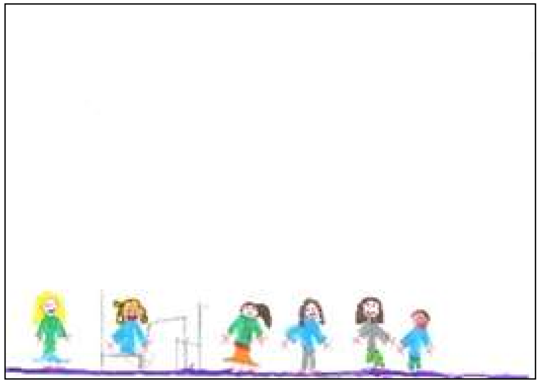

SF 11.1 | Bottom: *"These are me and my friends playing together in the sleeping room"*

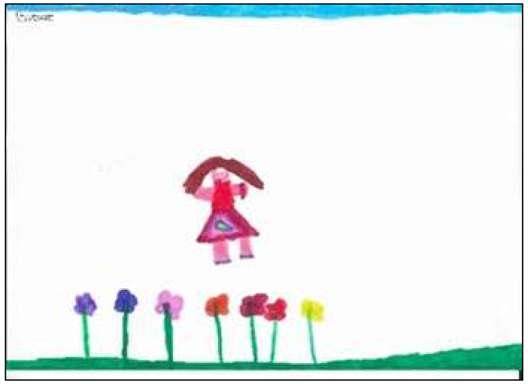

SF 11.2 | Center: *"Me in the school garden with flowers and the sky"*

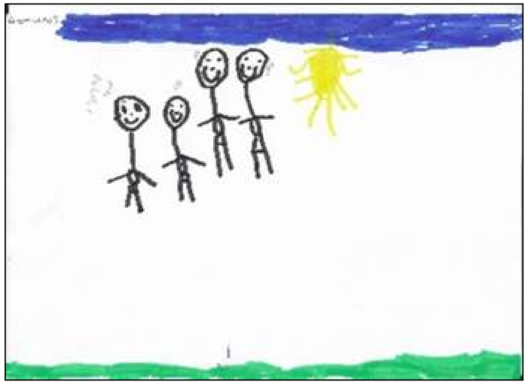

SF 11.3 | Top: *"I drew me, my brother Richard, mum and dad in the school garden"*

12. EMO_Climate

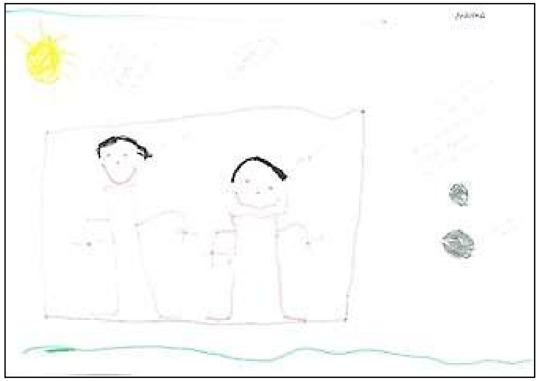

SF 12.1 | Positive:*"Me and my friend Jacopo inside the school looking at the snow flakes outside"*

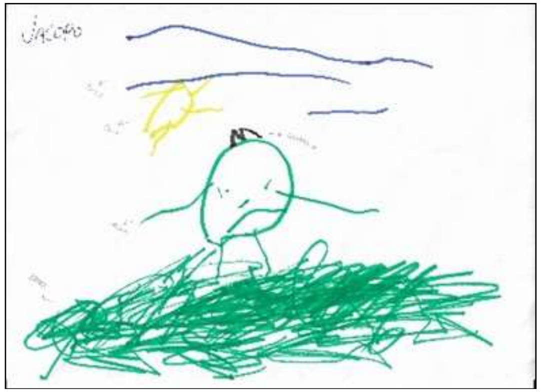

SF 12.2 | Negative: *"This is me in the school garden, with a hat on my head"*

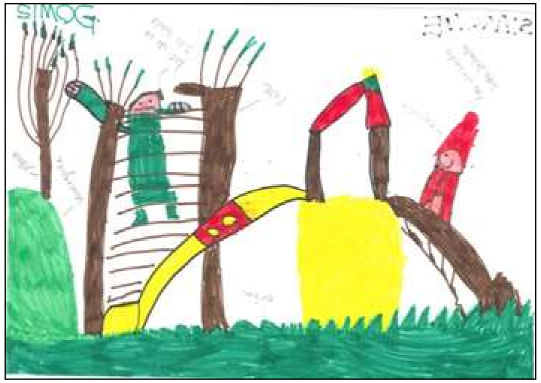

SF 12.3 | Mixed: *"This is me going on the slide and then this is the net that Lori goes on the ropes in the school garden"*

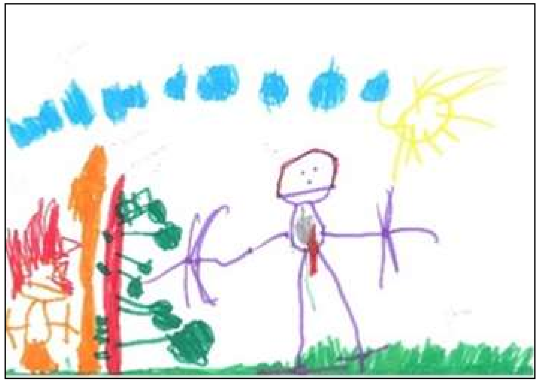

SF 12.4 | Neutral:*" These are me and nanny Monica in the school garden, with the school and a tree "*

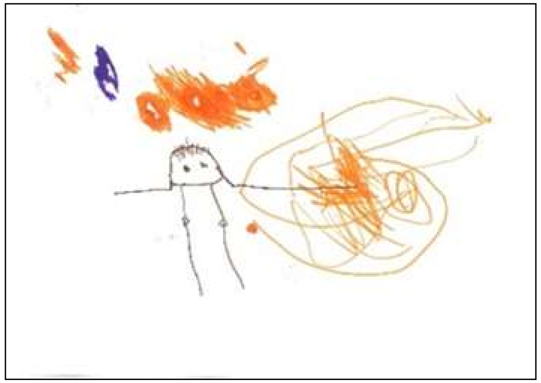

SF 12.5 | Not represented: *"This is me in the classroom"*

13. EMO_Archetypes

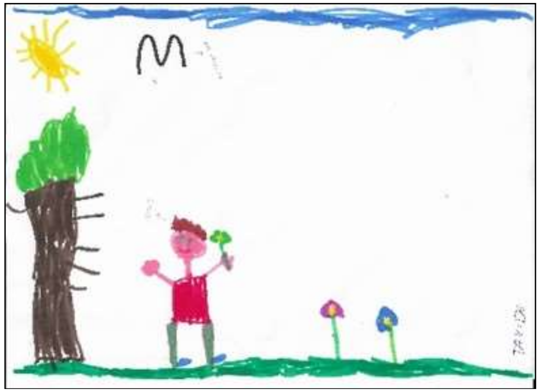

SF 13.1 | Land line, sky line, sun, trees flowers, animals: *"Me in the school garden picking flowers, with a tree and a bird"*

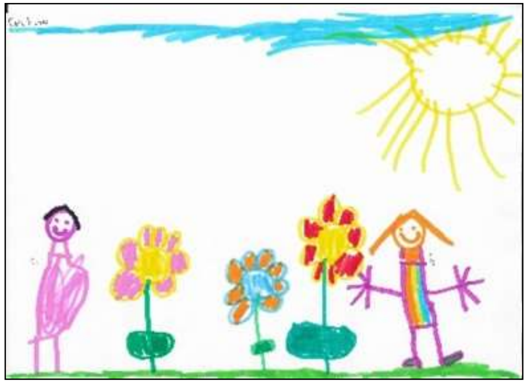

SF 13.2 | Land line, sky line, sun flowers: *"This is me playing in with my friend Matilde and the flowers"*

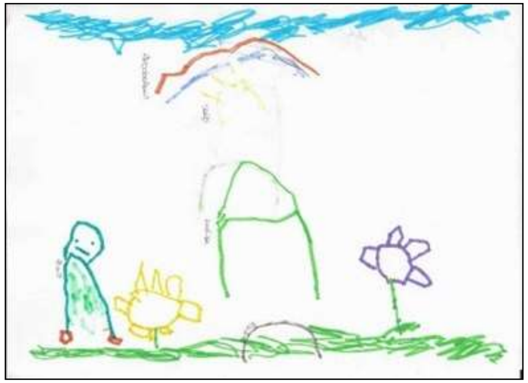

SF 13.3 | Land line, sky line, flowers, rainbow: *"This is me in the garden with the school, the flowers, the sky and the rainbow"*

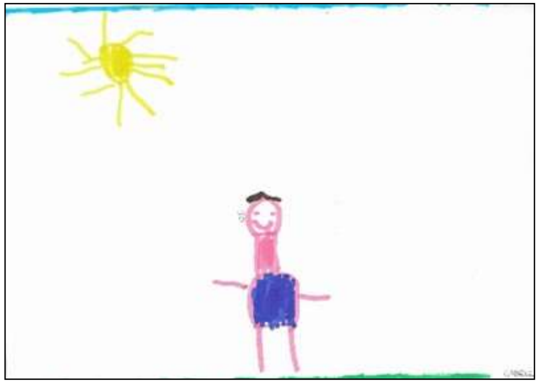

SF 13.4 | Land line, sky line, sun: *"This is me in the garden with the school, the flowers, the sky and the rainbow"*

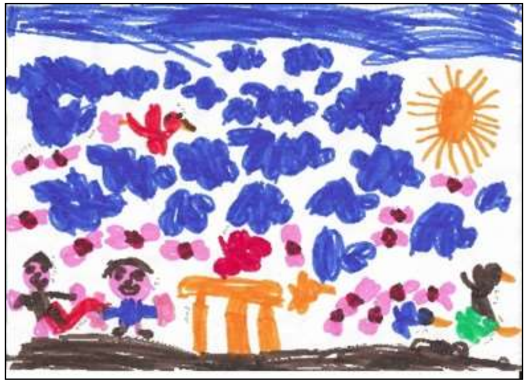

SF 13.5 | Land line, sky line, sun animals: *"This is me and my friend Tullio near the table in the garden, looking at birds and ladybugs"*

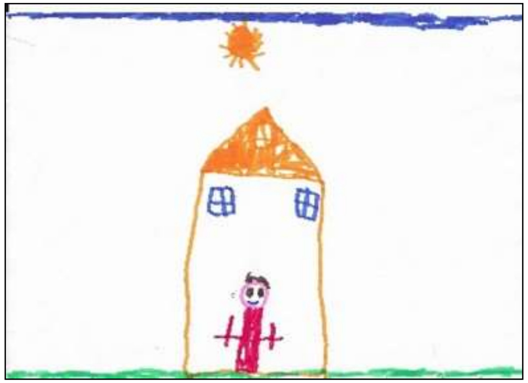

SF 13.6 | Land line, sky line, sun: *"This is me in the school with the garden outside"*

14. EMO_Colors_tone

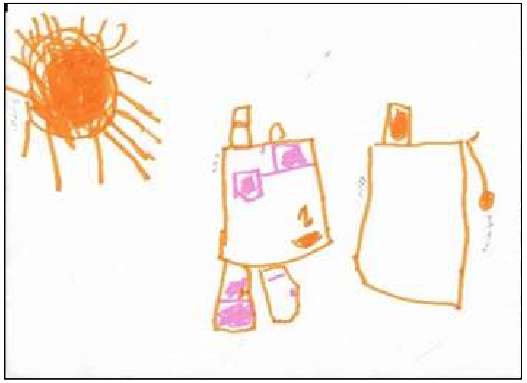

SF 14.1 | Warm: *" I drew the pretend play corner with the kitchen and the mirror, and the sun "*

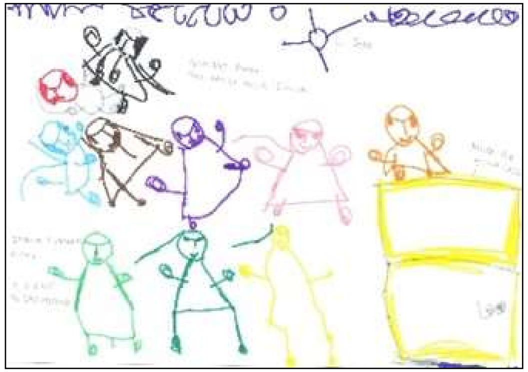

SF 14.2 | Both: *"This is me and my friends playing outside, near the school door"*

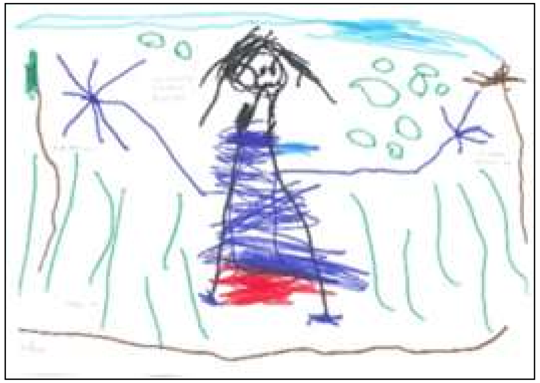

SF 14.3 | Cold: *"This is me in the garden with the grass, the sky and the clouds"*

15. EMO_Colors_variety

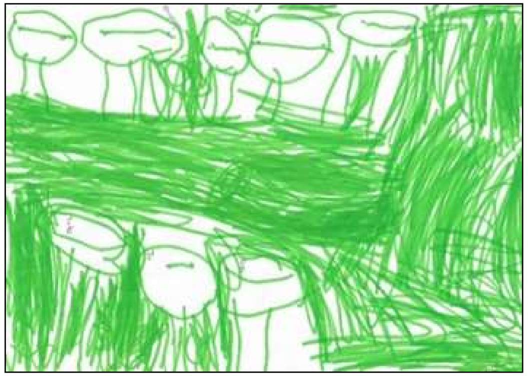

SF 15.1 | One color: *"I drew me, the teachers and my friends in the school garden mirror, and the sun"*

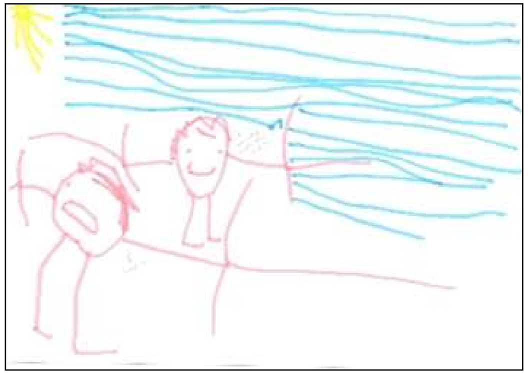

SF 15.2 | Up to four colors: *"This is me playing with my friend in the school garden"*

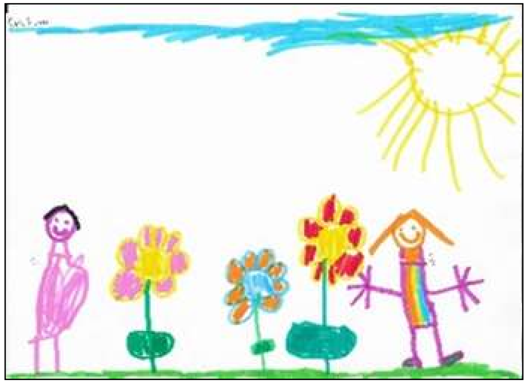

SF 15.3 | More than four colors: *"This is me in the garden with my friend Matilde"*

16. EMO_Position_Horizontal

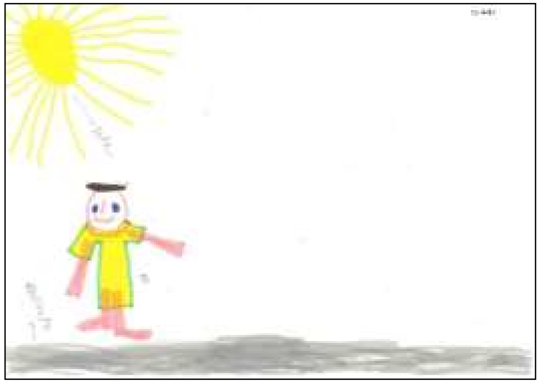

SF 16.1 | Left: *"It's me playing hide and seek on the cemented pavement outside"*

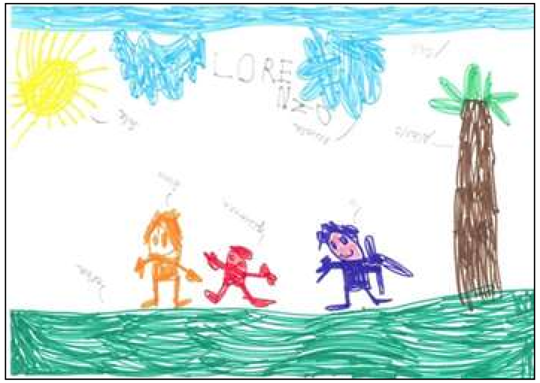

SF 16.2 | Center: *"Me and my friends playing in the school garden"*

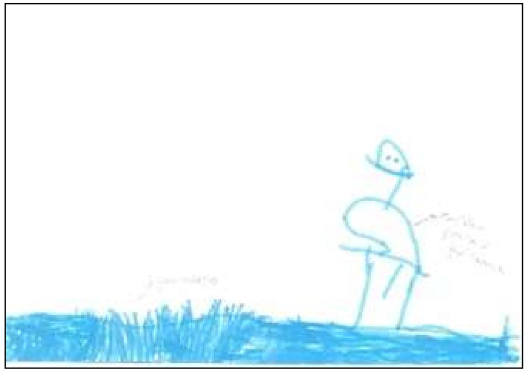

SF 16.3 | Right: *"That's me running in the corridor"*

17. EMO_ Position_Vertical

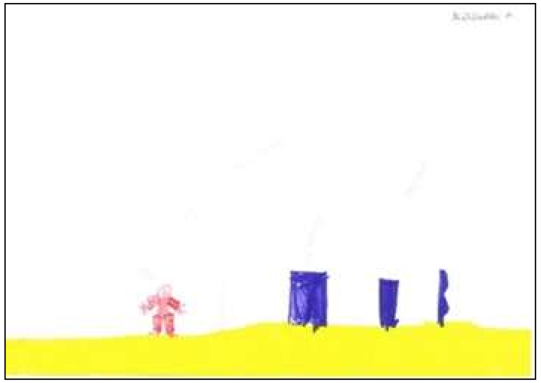

SF 17.1 | Bottom: *" This is me in the corridor with the foam blocks"*

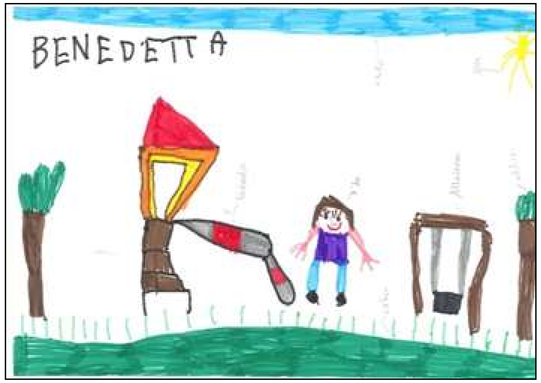

SF 17.2 | Center: *"I drew myself in the garden, I am going on the slide and there is also the swing"*

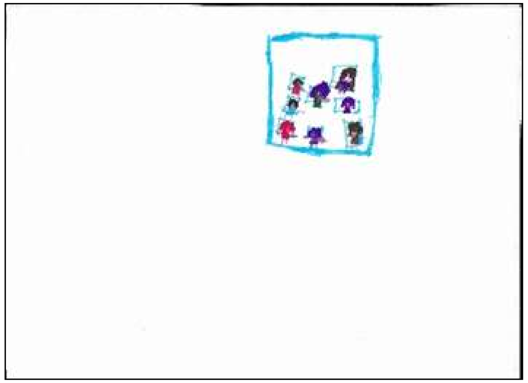

SF 17.3 | Top: *"This is my school, we're in the library"*

18. Not Relevant Drawings

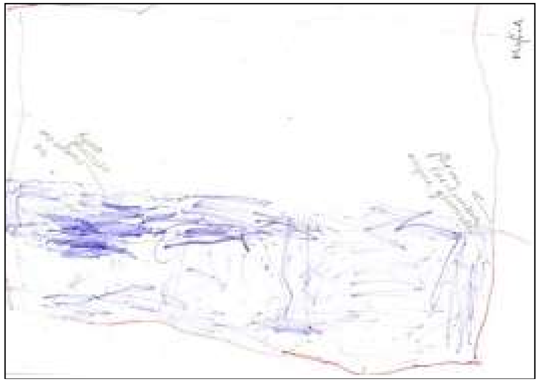

SF 18.1 | Not Relevant: *“I drew a river when the water never freeze"*

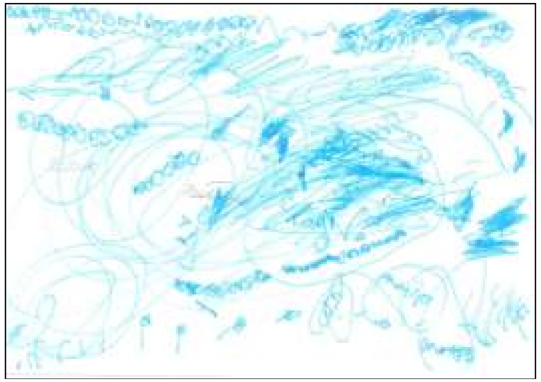

SF 18.2 | Not Relevant: *“I made a scribble"*

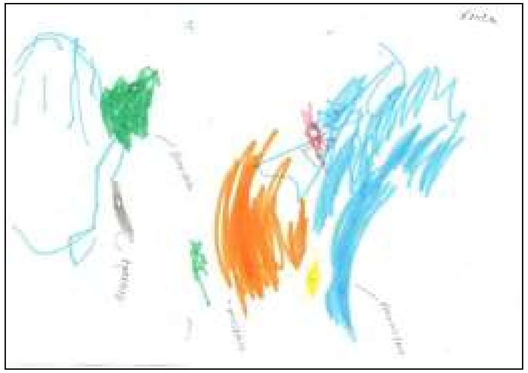

SF 18.3 | Not Relevant: *“I drew a monster, a ghost, a witch and a zombie”*
